# Gene expression in notochord and *nuclei pulposi*: a study of gene families across the chordate phylum

**DOI:** 10.1186/s12862-023-02167-1

**Published:** 2023-10-27

**Authors:** Rahul Raghavan, Ugo Coppola, Yushi Wu, Chibuike Ihewulezi, Lenny J. Negrón-Piñeiro, Julie E. Maguire, Justin Hong, Matthew Cunningham, Han Jo Kim, Todd J. Albert, Abdullah M. Ali, Jean-Pierre Saint-Jeannet, Filomena Ristoratore, Chitra L. Dahia, Anna Di Gregorio

**Affiliations:** 1 Hospital for Special Surgery, Orthopedic Soft Tissue Research Program, New York, NY 10021 USA; 2 Stazione Zoologica ‘A. Dohrn’, Villa Comunale 1, 80121 Naples, Italy; 3 Present Address: Molecular Cardiovascular Biology Division and Heart Institute, Cincinnati Children’s Research Foundation, Cincinnati, OH 45229 USA; 4 Department of Molecular Pathobiology, New York University College of Dentistry, New York, NY 10010 USA; 5 Hospital for Special Surgery, New York, NY 10021 USA; 6 Weill Cornell Medical College, New York, NY 10065 USA; 7 Department of Medicine, Columbia University Irving Medical Center, New York, NY 10032 USA; 8 Department of Cell and Developmental Biology, Weill Cornell Medicine, Graduate School of Medical Science, New York, NY 10065 USA

**Keywords:** *Ciona*, *Coronin*, Human, Mouse, Notochord, *Nucleus pulposus*, *Phip*, *Rgm*, *Rgs*, *Tmod*, *Xenopus*, Zebrafish

## Abstract

The transition from notochord to vertebral column is a crucial milestone in chordate evolution and in prenatal development of all vertebrates. As ossification of the vertebral bodies proceeds, involutions of residual notochord cells into the intervertebral discs form the *nuclei pulposi*, shock-absorbing structures that confer flexibility to the spine. Numerous studies have outlined the developmental and evolutionary relationship between notochord and *nuclei pulposi*. However, the knowledge of the similarities and differences in the genetic repertoires of these two structures remains limited, also because comparative studies of notochord and *nuclei pulposi* across chordates are complicated by the gene/genome duplication events that led to extant vertebrates. Here we show the results of a pilot study aimed at bridging the information on these two structures. We have followed in different vertebrates the evolutionary trajectory of notochord genes identified in the invertebrate chordate *Ciona*, and we have evaluated the extent of conservation of their expression in notochord cells. Our results have uncovered evolutionarily conserved markers of both notochord development and aging/degeneration of the *nuclei pulposi*.

**Supplementary Information:**

The online version contains supplementary material available at 10.1186/s12862-023-02167-1.

## Introduction

In all chordate embryos, the notochord is the main source of axial support and patterning cues [1–5]. In invertebrate chordates, *i.e.* tunicates and cephalochordates, the notochord persists throughout embryogenesis as the main supporting structure for the developing body. In vertebrates, the notochord undergoes segmentation as it is gradually replaced by the vertebral column, and its remnants are incorporated in the intervertebral discs (IVDs), where they form the centrally located *nuclei pulposi* (NP). The IVDs allow movement in between vertebrae, and evenly spread the mechanical loading on the vertebral bodies. The NP, with their gelatinous consistency, are critical for the function of the IVDs, and their alteration is intimately connected to IVD deterioration [6–8]. Because they are an integral component of the NP, notochord cells have emerged in recent years as a therapeutic avenue for human IVD regeneration [9].

Members of the tunicate subphylum are considered the invertebrate chordates most closely related to vertebrates [10], and indeed the tunicate notochord has been shown to share distinctive morphological and molecular traits with the vertebrate notochord (reviewed in [4, 11]. In the tunicate *Ciona*, the notochord consists of only 40 cells, arranged in a single row (*e.g.*, [12]). In the teleost zebrafish (*Danio rerio*), the notochord consists of two layers (inner and outer) of vacuolated cells [13]. In *Xenopus*, mouse, and humans, the notochord is composed of several cells that develop large vacuoles as this structure differentiates [1, 14–17]. In all these species, the notochord cells secrete abundant amounts of extracellular matrix proteins, which form a thick notochordal sheath [1, 15, 18–20].

Tunicate genomes have not undergone the extensive duplication events that have shaped the genomes of vertebrates [21, 22] and a considerable fraction of the genes that in vertebrates have originated composite gene families appear in single copy in *Ciona* and other tunicates, even though relevant examples of lineage-specific gene duplications have been reported [23–27]. Therefore, tunicates can serve as an informative point of reference for reconstructing the evolutionary origins of complex vertebrate gene families and for studies of conservation/divergence of gene expression patterns. We have previously assessed the conservation of notochord gene expression between two divergent tunicates, *Ciona robusta* and *Oikopleura dioica* [28]; in addition to that, we have identified counterparts of *Ciona* notochord genes in the mouse genome, and determined that the notochord expression observed in *Ciona* is mirrored by the majority of the mouse genes that we analyzed [29]. More recently, we have demonstrated that the cross-regulatory relationship that we uncovered in *Ciona* between two notochord transcription factors, Brachyury and Xbp1, is conserved in *Xenopus* [30].

Our previous studies show that hallmark notochord genes, such as *Brachyury* and *Sonic hedgehog*, are expressed by the postnatal NP cells of mouse IVDs [31, 32], providing a rationale for examining the expression of other genes that may be conserved and possibly have functional significance in the maintenance of these cells.

Here we first report the notochord expression of five *Ciona* genes, which were identified as part of our ongoing effort to characterize the *Ciona* notochord genetic toolkit. We used these five *Ciona* genes as a starting point to survey the expression of notochord genes that are present in single copy in tunicates and, through duplication events, have given rise to multigenic families in vertebrates. We began this analysis with a gene, *Pleckstrin-homology domain interacting protein* (*Phip*), which has remained in single copy in all the chordates analyzed here, and we expanded this study to genes that in vertebrates are part of increasingly larger families. *Phip* encodes for a protein that binds to the insulin receptor substrate 1 (Irs1) and is hypothesized to act as a link between Irs1 and the insulin receptor, thus modulating the insulin pathway [33, 34]; mice lacking Phip1, the main isoform of Phip, develop hypoglycemia and have a short lifespan [35]. In humans, a nonsense mutation in the *PHIP* gene has been recently linked to Chung-Jansen syndrome, which is characterized by obesity and developmental delay [36]. Another gene we analyzed, repulsive guidance molecule (*Rgm*), is present in single copy in *Ciona*, while in vertebrates is part of a multigenic family with four distinct subfamilies (*RgmA-D*), whose components, originally identified as key players in neuronal growth dynamics, have been associated with the development of numerous tissues and structures, and with their respective pathologies [37]. A larger gene family of interest for this study is the regulators of G-protein signaling (*Rgs*), because of their high degree of conservation across divergent chordates and their requirement for the timely inactivation of G-proteins [38]. The protein encoded by the *Ciona robusta Rgs6/7* gene is equally related to members of the Rgs6 and Rgs7 subfamilies, which in vertebrates are involved, in particular, in the control of phototransduction [39, 40].

In addition to the aforementioned genes, we followed across the chordate spectrum the expression of the orthologs of a member of the *Coronin* gene family, *Ciona Coronin-7* (*Coro7*). Coronins are actin-binding regulators of cytoskeletal remodeling, vesicle trafficking and cell motility [41, 42] and belong to the WD-repeat superfamily [43]; we also analyzed two *tropomodulin*/*leiomodin*-related *Ciona* genes, which provided insights into lineage-specific changes in notochord gene expression.

Lastly, in an effort to use information gathered in *Ciona* to shed light on gene expression during the evolutionary and developmental transition from notochord to NP, and to guide the identification of genetic markers of discopathies, we have analyzed the expression of orthologs of these *Ciona* notochord genes in both aging and degenerating mammalian NP.

## Results

### Identification of notochord genes in *Ciona*

We have previously reported the expression of orthologs of vertebrate notochord genes in *Ciona* [30, 44–47]. As a starting point for this study, we selected five genes expressed in the *Ciona* notochord for which the information on the expression in the vertebrate notochord was either fragmentary or lacking altogether.

**Fig. 1:**
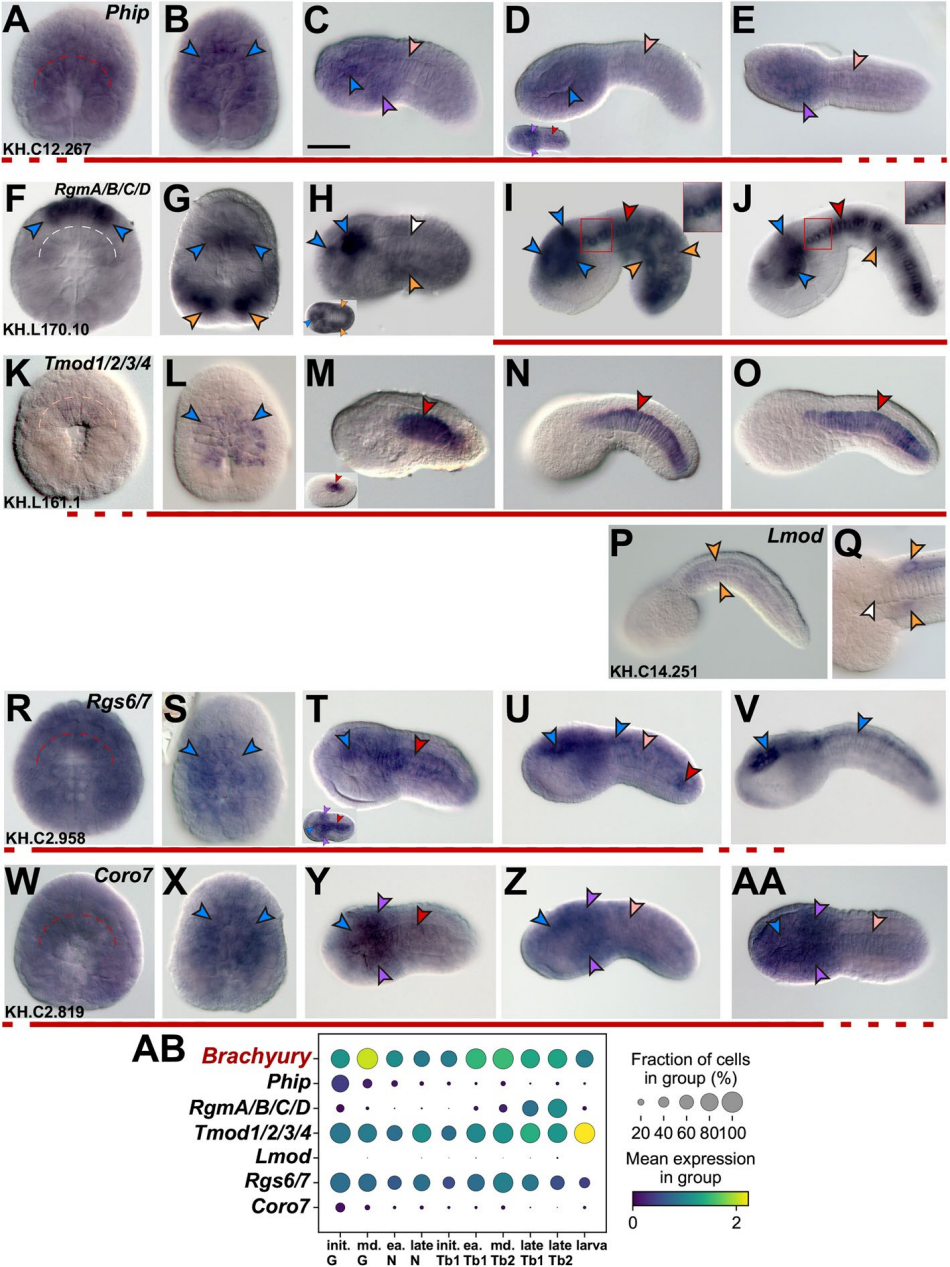
Identification of genes expressed during notochord development in *Ciona robusta*. *Ciona robusta* embryos ranging from early gastrula to late tailbud, hybridized in situ with antisense RNA probes specific for the genes reported on top of panels **A**, **F**, **K**, **P**, **R**, **W** (Table S1). Gene models are indicated in the bottom left corners. Insets in (**D**, **H**, **M**, **T**) show embryos at different developmental stages. Insets in (**I**, **J**) show a higher magnification view of the regions boxed in red in the main panels, to display staining in notochord cells, which is clearer in the trunk because it is not obscured by the staining in muscle cells. Stained territories are denoted by arrowheads, color-coded as follows: red, notochord; light pink, fading notochord staining; white, no detectable notochord staining; blue, CNS; purple, mesenchyme; orange, muscle. Red lines underneath the panels indicate the approximate time span of notochord expression throughout development. In (**A**, **F**, **K**, **R**, **W**) dashed curved lines delineate the location of notochord precursors. Scale bar: 50 µm. (AB) Dot plot summary of the published scRNA-Seq data [50] available for the genes in (A-AA), compared to the scRNA-Seq data available for *Ciona Brachyury*, which was used as a reference for notochord expression [51]. In the dot plot, each dot represents two values: the mean expression of each gene (visualized by color) and the fraction of cells expressing each gene (visualized by the size of the dot). The embryonic stages used by Cao et al. [50] to generate the scRNA-Seq dataset reflect only approximately the stages that we used for WMISH. Abbreviations: init., initial; md., middle; ea., early; G, gastrula; N, neurula; Tb, tailbud

Whole-mount in situ hybridization experiments revealed that expression of *Ciona Phip* (Fig. 1A-E; gene model: KH.C12.267; Table S1) is rather diffuse, and is detected throughout most of the embryo until gastrulation, resembling the expression pattern usually attributed to maternal transcripts [48, 49]. However, the notochord and neural precursors show a more intense signal (Fig. 1A,B), which at the tailbud stages becomes more localized to CNS and mesenchyme, while persisting at low levels in the notochord (Fig. 1C-E).

The *Ciona robusta* genome contains one identifiable member of the *Rgm* gene family, which encodes for a protein equally related to vertebrate Rgm proteins A-D, and has been therefore designated here as *RgmA/B/C/D* (Fig. 1F-J; gene model: KH.L170.10; Table S1). Starting from early developmental stages, this gene is specifically expressed in neural precursors (Fig. 1F) and by late gastrulation becomes detectable at high levels in muscle precursors as well (Fig. 1G). Expression in both territories is still detected in late neurulae (inset in Fig. 1H), however, in initial tailbuds, muscle expression begins to fade while expression in the CNS remains strong and becomes localized to different regions of the sensory vesicle (Fig. 1H). Finally, in later tailbud stages, expression in the CNS persists in the ventral region of the sensory vesicle, and weakens in muscle cells, while becoming detectable in the notochord (Fig. 1I,J). The expression of this gene in muscle and sensory vesicle is also reported in the Aniseed expression database (https://www.aniseed.fr) [52].

We also selected for this analysis two *Ciona* genes related to vertebrate *tropomodulin* genes, which we indicate here as *Tropomodulin1/2/3/4* (*Tmod1/2/3/4*) and *Leiomodin* (*Lmod*). The reason for using this nomenclature is that we observed that although the predicted proteins for both genes are related to tropomodulins from other species, the putative *Ciona* Lmod protein contains in its C-terminal a WASP-Homology 2 (WH2) actin-binding domain and a short proline-rich region, which are characteristic features of leiomodins from other species [53, 54].

*Tmod1/2/3/4* (Fig. 1K-O; gene model: KH.L161.1; Table S1) is first detected in invaginating notochord precursors (Fig. 1K) and is transiently expressed in neural precursors during late gastrulation (Fig. 1L); the hybridization signal increases considerably at the time of neurulation (inset in Fig. 1M) and remains intense and notochord-specific throughout the tailbud stages (Fig. 1M-O). On the other hand, *Lmod* (Fig. 1P,Q; gene model: KH.C14.251; Table S1), is detectable by WMISH only in muscle cells of late tailbuds (Fig. 1P,Q); this result is consistent with reports of muscle-specific or muscle-predominant expression of leiomodins [55].

Next, we analyzed the expression of a member of the *Rgs* gene family, which we named *Rgs6/7* because it is orthologous to both *Rgs6* and *Rgs7* genes identified in vertebrates (see below). In early embryos, at the 16-cell stage, expression of *Ciona Rgs6/7* (gene model: KH.C2.958; Table S1) had been reported in all blastomeres [56]. We found that although *Rgs6/7* remains widely expressed during early embryogenesis, in a pattern that is suggestive of maternal expression (Fig. 1R), by late gastrulation its expression begins to fade from the precursors of the epidermis (Fig. 1S). In initial tailbuds, *Rgs6/7* is detected in notochord and mesenchyme cells, as well as in the sensory vesicle (Fig. 1T). Around the mid-tailbud stage, expression in the notochord decreases, persisting mainly in cells of the secondary notochord, while expression in the CNS increases in intensity and broadens to encompass the entire sensory vesicle and the nerve cord, which extends throughout the length of the tail (Fig. 1U). In late tailbuds, expression in notochord cells falls below detection, while expression in the CNS increases further (Fig. 1V).

*Ciona Coronin7* (*Coro7*, Fig. 1W-AA; gene model: KH.C2.819; Table S1) displays a diffuse expression at early and late gastrula stages (Fig. 1W,X); by the initial tailbud stage, the hybridization signal becomes refined to sensory vesicle and mesenchyme, and persists in intercalating notochord cells, although at lower levels compared to the other territories (Fig. 1Y). Expression is reduced but still detectable in notochord cells throughout the mid-tailbud stages (Fig. 1Z,AA).

Even taking into account possible differences in the experimental conditions that influence embryonic staging (*e.g.*, temperature, incubation time), the results of our WMISH are mostly in agreement with published single-cell RNA-sequencing (scRNA-Seq) data [50] (Fig. 1AB). In particular, both results indicate that while *Tmod1/2/3/4* is strongly expressed in notochord cells at all stages analyzed (Fig. 1K-O, 1AB), expression of *Lmod* is either undetectable or negligible (Fig. 1P,Q,AB).

### Identification of vertebrate orthologs of *Ciona* notochord genes through phylogenetic analyses

In order to identify vertebrate orthologs of the *Ciona* notochord genes selected for this study (Fig. 1), we carried out phylogenetic analyses for the *Phip*, *Rgm*, *Tmod*, and *Coronin* families, employing a manually curated database of protein sequences (Supplementary files 1–5).

*Phip* genes (also known as *Brdw3*) have remained in single copy in the species analyzed in this study. We assessed their conservation by aligning Phip protein sequences selected from chordates and other metazoan taxa (Fig. S1; Supplementary file S1). As expected, the highest degree of sequence conservation was found in the WD-repeat region and in the bromodomain (highlighted in Fig. S1).

**Fig. 2:**
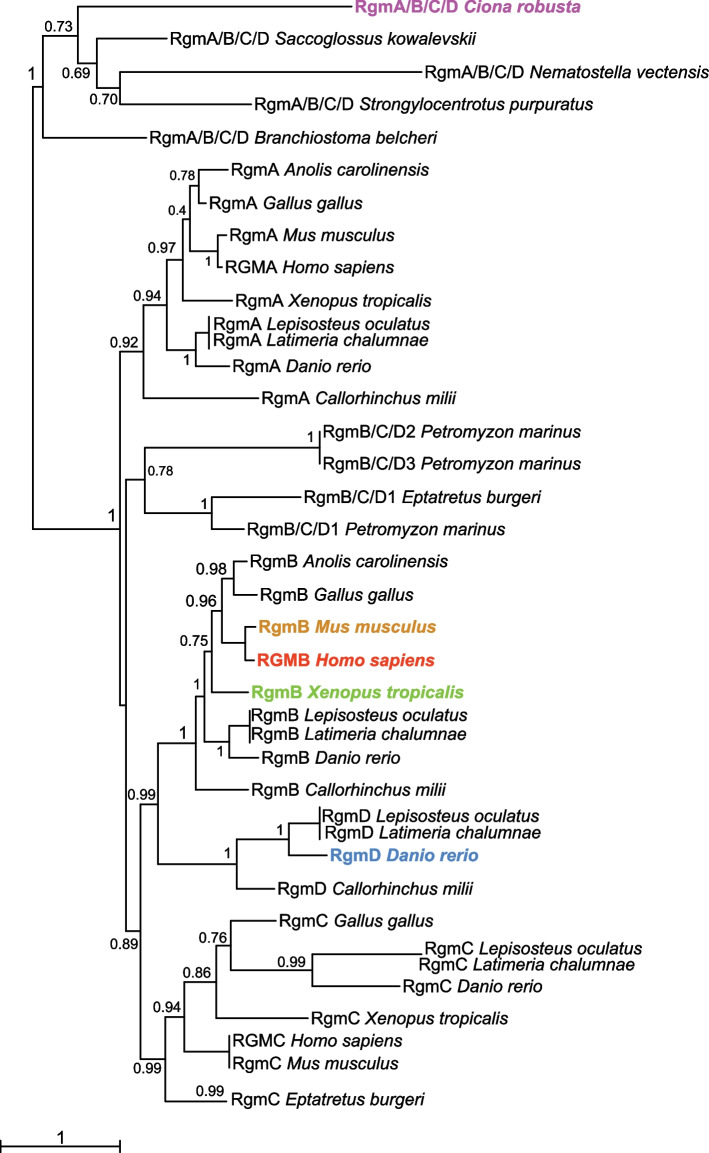
Phylogenetic reconstruction of the evolutionary relationships within the *Rgm* gene family. ML phylogenetic tree showing the relationships among members of different Rgm classes. Proteins encoded by genes present in invertebrates in single copy and equally related to the RgmA-D classes have been indicated as RgmA/B/C/D. RgmA, RgmB, and RgmC were found in all the vertebrates analyzed in this study, while *RgmD* genes have only been reported, thus far, in teleosts and cartilaginous fish. Distinct colors highlight the family members analyzed in this study. Values reported at the branching points indicate replicates obtained using the aLRT method

It was previously reported that invertebrate chordates possess a single-copy *Rgm* gene [57]; the maximum likelihood (ML) phylogenetic tree that we obtained for the *Rgm* family shows the relationship between the single-copy *Rgm* gene present in *Ciona* and in the non-chordate invertebrates selected for this analysis, which we termed *RgmA/B/C/D* (Fig. 2). Most vertebrate genomes contain three paralogous groups of *Rgm* genes: *RgmA*, *RgmB* (also known as *Dragon*) and *RgmC* (also known as *hemojuvelin*/*hjv*, or *hfe2*). Our phylogenetic reconstruction indicates that a fourth paralogous group of this gene family, *RgmD*, which thus far has only been reported in teleosts [58] and in cartilaginous fish (*C. milii*, Fig. 2), is closely related to the *RgmB* genes of tetrapods (Fig. 2). The close relationship between *RgmA* and *RgmB* paralogs had been previously reported, and is corroborated by the synteny analysis of their genomic surroundings; in particular, in syntenic regions of different chordates *RgmB* is linked to *Dnajb5* (*Hsp40*) [58]. Remarkably, the linkage of *Rgm* to *Dnajb5* is present in *Ciona* as well as in *Gasterosteus aculeatus* (stickleback), *Xenopus* and mouse [58]. We updated this earlier report using the latest version of the *Ciona robusta* genome [59, 60], traced these markers to the latest version of the human genome, and identified additional conserved markers in vertebrate genomes relevant to this study (Fig. S2). Of note, we found that the clustering of a *RgmA/B/C/D* gene with genes encoding chromodomain helicase DNA-binding proteins (*chd*), which are conserved neighbors of vertebrate *RgmA* and *RgmB* genes [58], is present in the genome of the hemichordate *Saccoglossus kowalevskii* (Fig. S2). We also found additional genes that are maintained in the proximity of *RgmA* and *RgmB* genes of vertebrates (Fig. S2, S3), a finding that reinforces the close relationship between *RgmA* and *RgmB* paralogs. Accordingly, our synteny analysis of vertebrate *RgmC* genes indicates a considerable difference between the genomic surroundings of these paralogs and those of the *RgmA* and *RgmB* genes (Fig. S3). None of the conserved neighbors of the *RgmA-C* genes seems to be present in the proximity of the *RgmD* paralogs in any of the species that we surveyed (Fig. S4). Most of the genes neighboring *RgmD* in fish genomes are maintained on the same chromosomes in the tetrapods analyzed here, despite the absence of *RgmD* in tetrapods (Fig. S4).

**Fig. 3:**
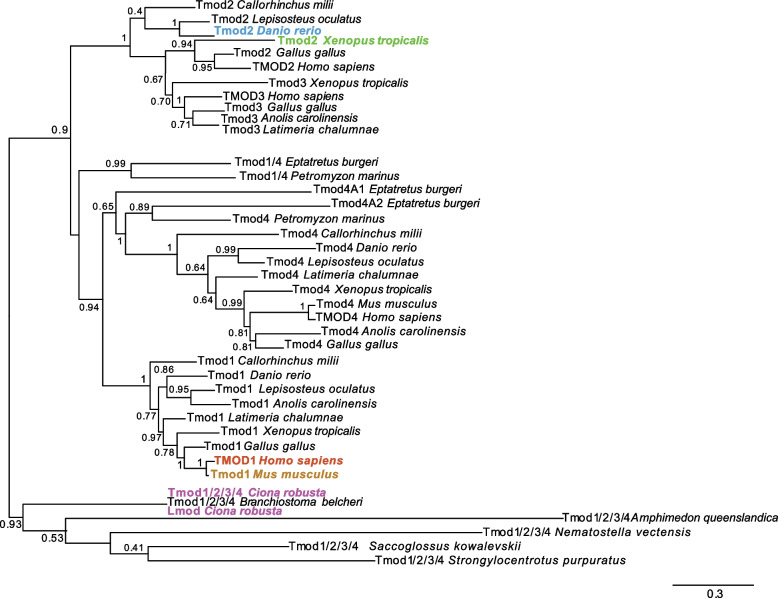
Phylogenetic reconstruction of the evolutionary relationships within the *Tmod* gene family. ML phylogenetic tree displaying the relationships among members of different Tmod classes. Proteins encoded by genes present in invertebrates in single copy and equally related to the 1–4 classes have been tentatively indicated as Tmod1/2/3/4. Tmod1, Tmod2, Tmod3 and Tmod4 have been found in all the vertebrates analyzed here. Distinct colors highlight the family members analyzed in this study. Values reported at the branching points indicate replicates obtained using the aLRT method

A phylogenetic reconstruction of the *tropomodulin* family is shown in Fig. 3, and indicates that the *Tmod2* and *Tmod3* subfamilies are closely related (Fig. 3), which is consistent with previous reports and with the clustered arrangement that these genes have in mammals and chickens [61]. The zebrafish genome contains only one of these two paralogs, which is commonly referred to as *tmod2* (Fig. 3), although it has been argued to be more closely related to *tmod3* [61]. We carried out a comparative study of the genomic surroundings of *Tmod2* and *Tmod3* genes across vertebrates, and found that *Callorhinchus milii* (elephant shark), *Latimeria chalumnae* (coelacanth), *Lepisosteus oculatus* (spotted gar) and stickleback also contain *Tmod2/Tmod3* genes in single copy, and that these genes are all located within genomic contexts comparable to those of the *Tmod2* and *Tmod3* genes found in tetrapods (Fig. S5).

**Fig. 4:**
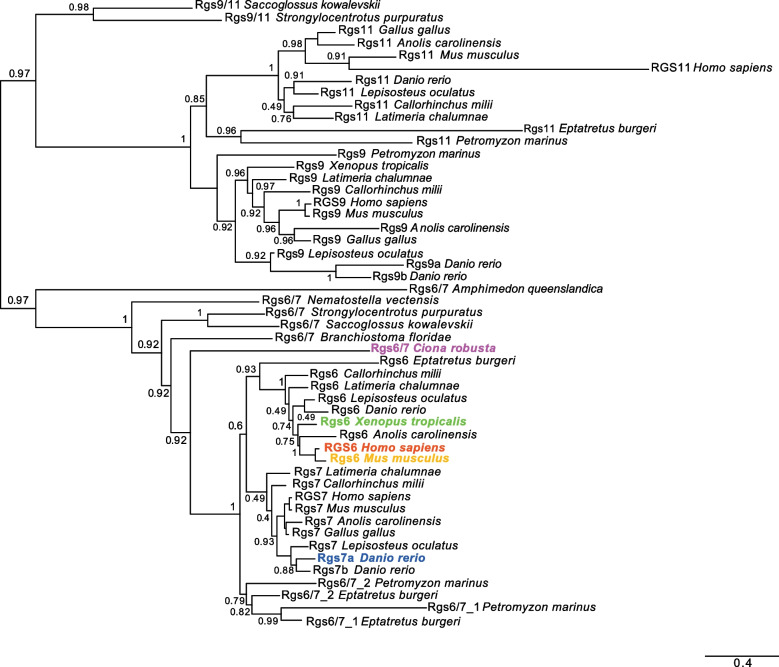
Phylogenetic reconstruction of the evolutionary relationships between proteins of the Rgs6/7 and Rgs9/11 subfamilies. A global phylogenetic study of the Rgs proteins found in *Ciona robusta* is available [38]. This ML phylogenetic tree is centered on the Rgs6/7 subfamily, since the expression of some of its members has been studied here, and on the Rgs9/11 subfamily, because of its close relationship to the Rgs6/7 subfamily. Distinct colors highlight the family members whose expression was analyzed in this study. Values at the branches indicate replicates obtained using the aLRT method

At least 21 *Rgs* genes have been reported in mouse [62], and in the human genome individual and/or tandem duplications of several members of this family have generated 20 canonical RGS proteins and 19 RGS-like proteins [63]. A complete phylogenetic study of the Rgs proteins found in *Ciona robusta* was already available [38], and *Rgs6*, *Rgs7*, *Rgs9* and *Rgs11* had been suggested to share a common evolutionary origin [38, 62]; therefore, we focused our phylogenetic analysis on members of these groups (Fig. 4). In particular, the presence of two *rgs7* orthologs in zebrafish suggests that they have stemmed from the teleost-specific WGD event [64, 65], and prompted us to analyze the expression of *Danio rgs7* genes.

**Fig. 5:**
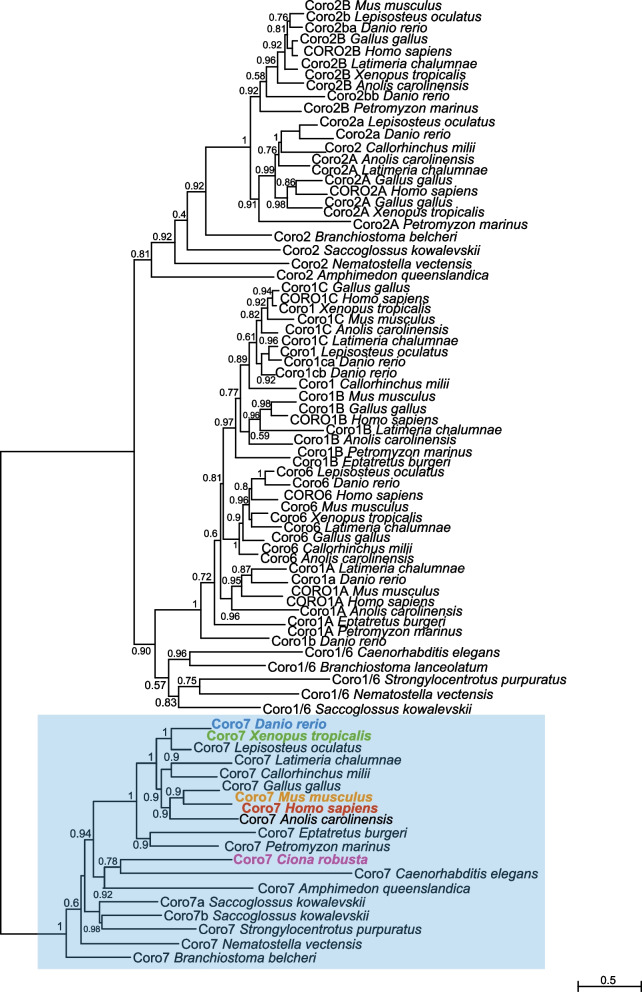
Phylogenetic reconstruction of the evolutionary relationships within the *Coronin* gene family. ML phylogenetic tree of the Coronin (Coro) family. The Coro7 subfamily is highlighted by a light blue box. Distinct colors indicate the family members whose expression was analyzed in this study. Values reported at the branching points indicate replicates obtained using the aLRT method

The evolutionary origins of *coronin* genes can be traced back to either a single gene [66] or two related genes [67], which expanded to give rise to at least 723 coronin proteins grouped into different classes [43]. Our ML phylogeny highlighted the relationships among the members of four subfamilies, *Coro1*, *Coro2*, *Coro6* and *Coro7* (Fig. 5). Although class-4 coronins have been identified from different eukaryotic species, they have undergone lineage-specific events that caused gain/loss of their PH and gelsolin protein domains [67]. This high variability, along with the paucity of coronin-4 homologs and the changes in nomenclature of some of these genes, prevented the inclusion of this subfamily in this analysis. With the notable exception of the hemichordate *S. kowalevskii*, members of the *Coro7* subfamily seem to have remained in single copy, while members of the other subfamilies are duplicated in vertebrates (Fig. 5). Remarkably, our synteny analysis showed that one of the *Coro7* genes of *S. kowalevskii* is linked to *Dnaja3*; in *Ciona robusta*, this gene is not adjacent to *Coro7*, but is located on the same chromosome (Fig. S6). One of the introns of *Ciona Coro7* contains a coding region whose predicted product is a transmembrane protein related to both fibronectin leucine-rich transmembrane protein 1 (flrt1) and vasorin (vasn). Vasorin acts as a negative regulator of TGF-beta signaling [68] and appears embedded within the *Coro7* transcription unit in all the genomic loci that we analyzed, except in the case of the *Danio* genome (Fig. S6). Other genes found on the same *Ciona* chromosome as *Coro7*, and conserved in the corresponding vertebrate loci, are *Pam16* and *Hmox* (Fig. S6). Interestingly, in human and in other vertebrate genomes *Coro7* and *Pam16* are located in close proximity and arranged in tandem orientation, and form a naturally occurring read-through transcription unit [69]. We found that this arrangement is not present in *Saccoglossus* and *Ciona*; *Saccoglossus Pam16* is located on a sequence scaffold different from those of *Coro7a* and *Coro7b*, while in *Ciona* these genes are very distant, even though they are located on the same chromosome (Fig. S6). In spotted gar, zebrafish, and all vertebrates analyzed here, *Coro7* and *Pam16* are adjacent and in tandem orientation (Fig. S6), which suggests that this read-through transcription unit formed early during vertebrate evolution.

### Expression of zebrafish orthologs of *Ciona* notochord genes

**Fig. 6:**
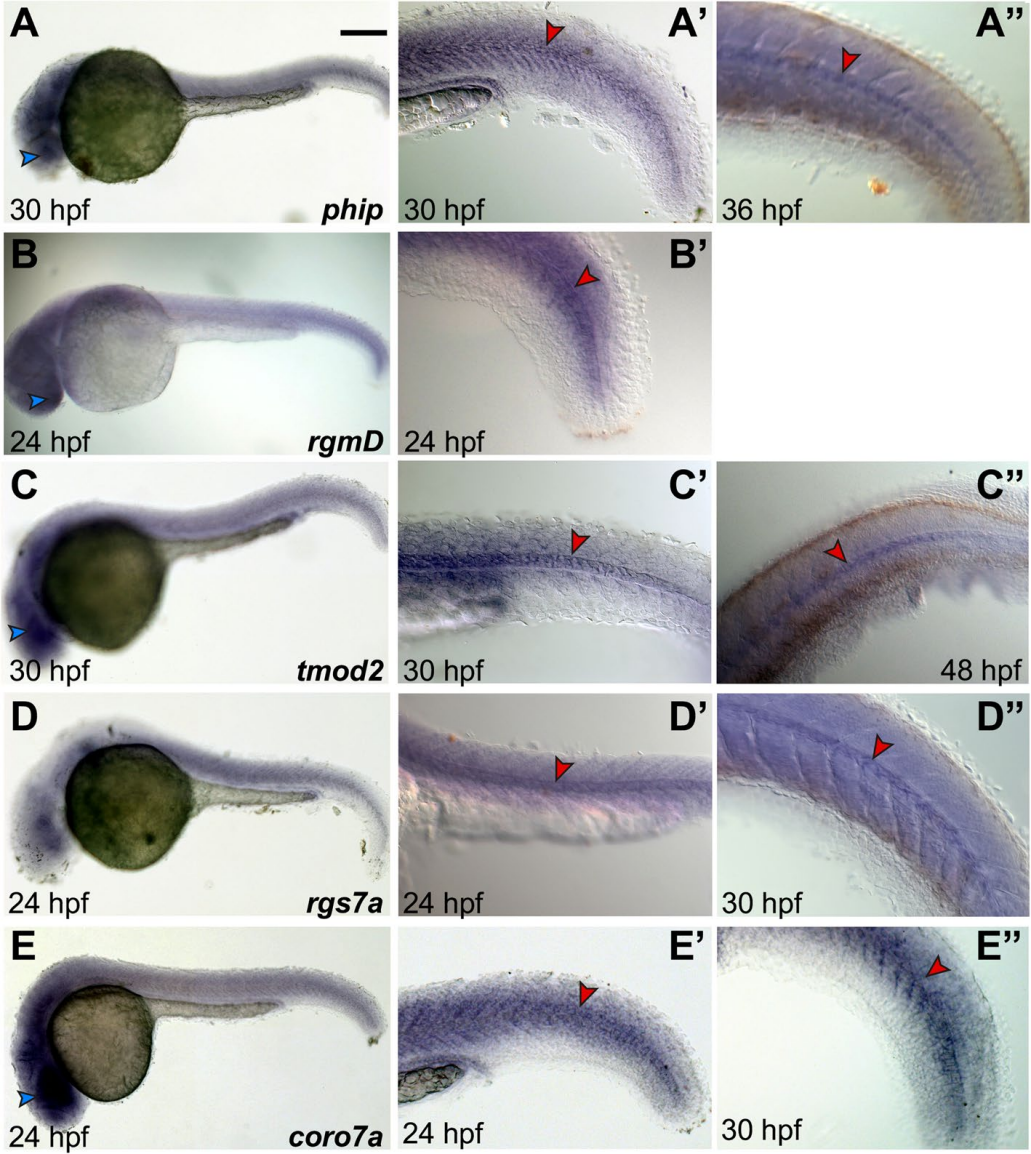
Expression of *phip, rgmD, tmod2, rgs7a* and *coro7a* in *Danio rerio*. **A**-**E** Whole-mount zebrafish embryos at the stages indicated at the bottom of each panel, hybridized in situ with probes specific for the genes indicated on the bottom right of panels A-E. (**A’**-**E”**) Close-ups of the tails of stained embryos, either magnified from (A-E) or acquired from representative embryos from the same batch as those in (A-E). Arrowheads are color-coded as follows: blue, nervous system; red, notochord. All panels show lateral views, anterior to the left. Scale bar: 150 µm

To assess the conservation of notochord expression of vertebrate genes related to the *Ciona* notochord genes described above, we analyzed *Danio* genes that were selected on the basis of phylogenetic analyses and database searches. We found that *Danio phip* is expressed in notochord and nervous system at 30 hpf (Fig. 6A,A’) and 36 hpf (Fig. 6A”).

The *rgm* family in zebrafish consists of four genes, *rgma*, *rgmb*, *rgmc* and *rgmd* (Fig. 2). While *rgma* is expressed predominantly in floor plate, developing midbrain and hindbrain, skeletal muscle and notochord [70–73], *rgmb* is expressed in several domains of the nervous system and in developing muscle, with faint expression in the notochord during early developmental stages [57, 74], and *rgmC* is strongly expressed in the notochord and its flanking somites [72]. Hence, here we investigated the poorly characterized zebrafish *rgmd*. According to expression patterns retrieved from the Zfin database (zfin.org; [75]), expression of this gene is widespread throughout the embryo from 10–13 somites to 14–19 somites stage [74]. Our WMISH results show an unlocalized signal in the head and a similar diffuse expression throughout the tail (Fig. 6B). However, by 24 hpf, we detected a considerable increase in notochord expression (Fig. 6B,B’).

With respect to the *tmod* family, zebrafish only contains three of the four *tmod* genes reported in mammals, *tmod1*, *tmod2* and *tmod4* [76] (Fig. 3). Since *tmod1* and *tmod4* are well-characterized genes expressed in muscle cells and involved in muscle development [76, 77], here we analyzed *tmod2* and found that this gene is expressed in notochord cells at 30 hpf and 48 hpf (Fig. 6C-C”).

The zebrafish genome contains three orthologs of *Ciona Rgs6/7*: *rgs6*, *rgs7a* and *rgs7b* (Fig. 4). Expression of both *rgs6* and *rgs7a* had been reported in various regions of the nervous system, and expression of *rgs7a* had also been reported in notochord cells (zfin.org; [75]). Using specific probes for each gene, we detected expression of *rgs7a* in notochord at both 24 hpf and 30 hpf (Fig. 6D-D”). No detectable hybridization signal was observed for *rgs7b* at the stages that we analyzed; accordingly, RNA-Seq data indicate that the expression levels of this gene during early embryogenesis peak at the 128-cell and 1k-cell blastula stage, drop at the dome stage, and remain low until the late larval stages [78].

*Danio coro7* is strongly expressed in various structures of the head, and in particular in the developing nervous system (Fig. 6E); expression becomes detectable in the notochord by 24 hpf (Fig. 6E’) and persists at later stages (30 hpf; Fig. 6E”).

### Expression of *Xenopus* orthologs of *Ciona* notochord genes

**Fig. 7:**
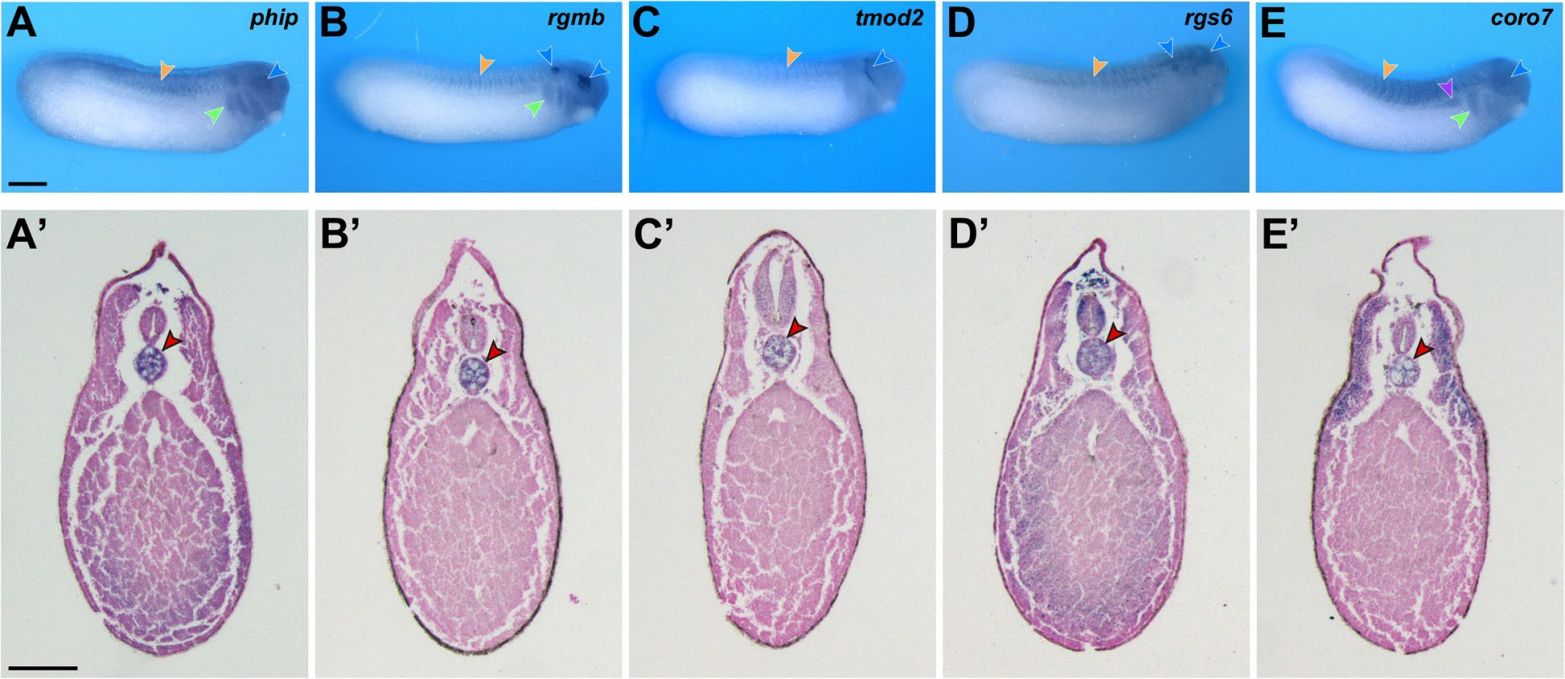
Expression of *phip, rgmb, tmod2, rgs6* and *coro7* in *Xenopus laevis.* **A**-**E** Whole-mount in situ hybridization of NF stage 28 embryos. Lateral views, anterior to the right, dorsal on top. (**A’**-**E’**) Transverse sections of NF stage 32 embryos. Dorsal is on top. Arrowheads color code: red, notochord; blue, nervous system; orange, somites; green, branchial arches; violet, pronephros. Scale bars: 500 µm (A); 200 µm (A’)

To follow the expression of the genes of interest in an additional vertebrate clade, we cloned the *Xenopus laevis* orthologs of *Ciona* notochord genes and analyzed their expression by WMISH. With the exception of one brief report describing the diffuse expression of *tmod2* at NF stage 30 [79], the expression of *Xenopus phip, rgmb*, *tmod2*, *rgs6* and *coro7* had not been previously reported. We found that at the tailbud stage (NF stage 28), *Xenopus phip* is enriched dorsally in the trunk, in the head region, the optic vesicles and the branchial arches (Fig. 7A), whereas *rgmb* shows strong signal in the optic and otic vesicles, with weaker expression in the branchial arches (Fig. 7B). Additionally, *tmod2* is expressed around the developing eye, in a region corresponding to the prospective trigeminal nerve (Fig. 7C). *rgs6* is detected in the brain and the otic vesicles and shows more diffuse expression in the somites (Fig. 7D), while *coro7* is expressed dorsally in the developing somites, the pronephros, the optic vesicles and the branchial arches (Fig. 7E).

To assess the expression of these genes in deeper tissues we performed transverse sections on NF stage 32 embryos, and we found that all five genes are expressed in the *Xenopus* notochord (Fig. 7A’-E’).

### Expression of mouse and human orthologs of *Ciona* notochord genes

To test the conservation of the expression of *Ciona* notochord genes in the notochord and in notochord descendant NP cells in mouse and human, we used the results of the phylogenetic analyses (Figs. 2, 3, 4 and 5) to identify candidate orthologs of *Ciona* notochord genes in the *Mus musculus* and *Homo sapiens* genomes. With the exception of *Rgmd* genes, which thus far have only been described in cartilaginous fish and teleosts, all members of the *Rgm* family were identified in mouse (*Rgma*, *Rgmb*, and *Rgmc/Hjv/Hfe2*) and human (*RGMA, RGMB,* and *RGMC/HJV/HFE2*) (Fig. 2). Similarly, the *Tmod* family is fully represented in both mouse (*Tmod1-4*) and humans (*TMOD1-4*) (Fig. 3), and conserved members of the *Rgs* subfamilies *6*, *7*, *9* and *11* (Fig. 4), and *Coronin* subfamilies, including *Coronin7* orthologs (Fig. 5), are present in both mouse and humans. Previously, expression of mouse *Coro7*, also known as *Pod-1*, had been reported in the developing brain and in parts of the immune system [80].

Next, we analyzed the expression of the genes selected for this study in various stages and tissues, during embryonic and post-natal development. Published single-cell RNAseq data obtained in mouse embryos at different stages of gastrulation (E-MTAB-6967; [81]) at E6.5 (primitive streak stage), E7.25 (node/notochord formation), and E8.5 (fully formed notochord) indicated that *Phip, Coro1b, Coro1c,* and *Tmod3* were ubiquitously expressed in all embryonic structures, including primitive streak and notochord; moreover, expression of *Rgmb* and *Tmod2* was particularly robust in the notochord of E8.5 embryos (Fig. S7).

**Fig. 8:**
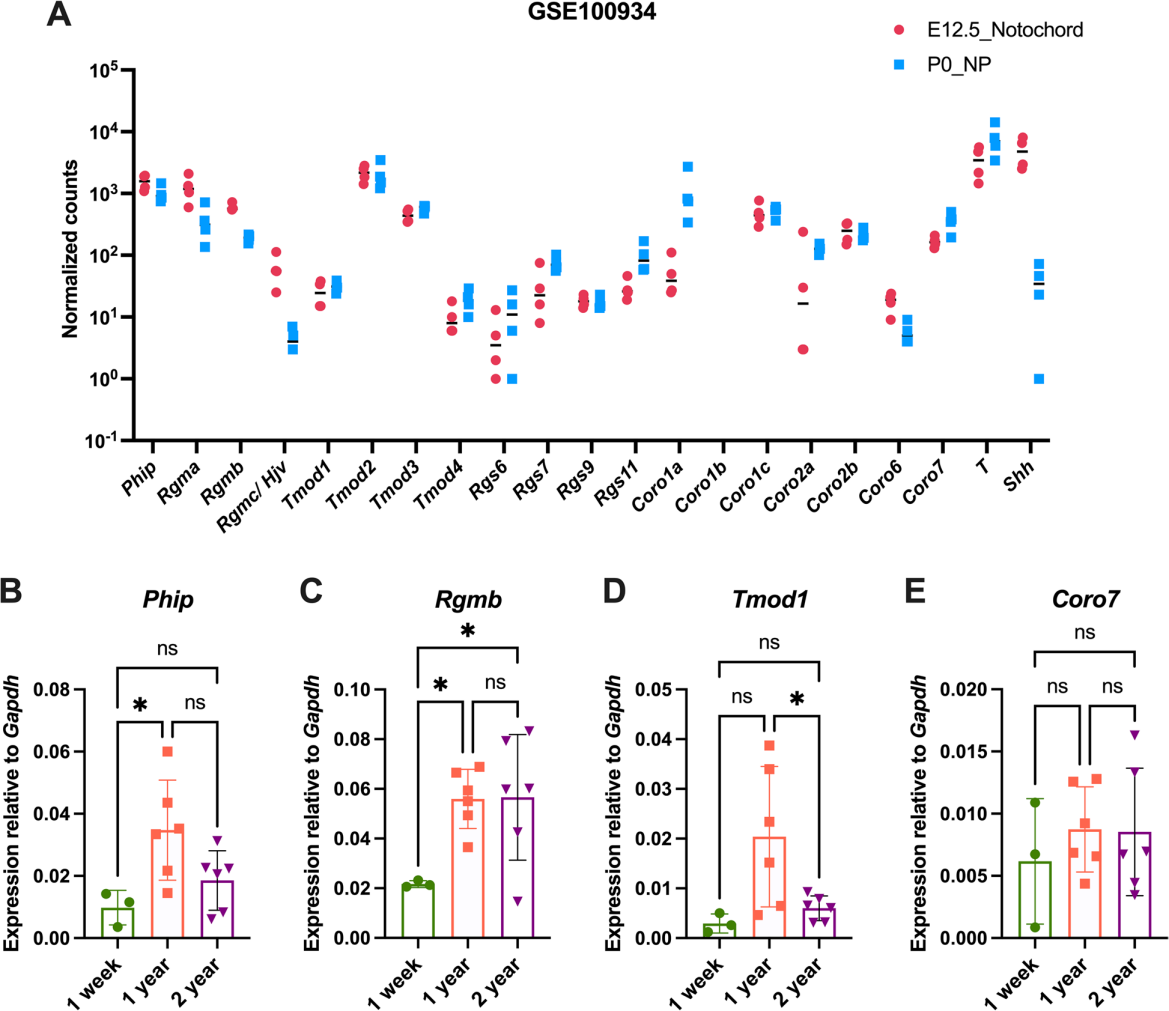
Gene expression analysis in mouse *nuclei pulposi*.** A** Normalized read counts in E12.5 notochord and P0 NP cells from the GSE100934 dataset [82]. **B-E** Multiplex qPCR analysis showing the expression of *Phip* (B), *Rgmb* (**C**), *Tmod1* (**D**)*,* and *Coro7* (**E**) relative to *Gapdh* in one-week (*n* = 3), one-year (*n* = 6) and two-year (*n* = 6) old mouse NP cells from lumbar IVDs of wild-type mice. Results are presented as scatter dot plots with mean and SD for each cohort. The qPCR results plotted in (**B-E**) were analyzed by ordinary one-way ANOVA followed by Tukey’s multiple comparisons test. * = *p* < 0.05

Next, using published bulk-RNAseq data from murine E12.5 notochord and post-natal day 0 (P0) NP cells (GSE100934; [82]), we found expression of all the orthologs of *Ciona* notochord genes at both stages (Fig. 8A), with the exception of *Coro1b*, which had zero reads in the database that we used. Expression of *RgmC/Hjv* (reported as *RgmC* in Fig. 2) is considerably reduced in the transition from notochord to NP cells (Fig. 8A). A reduction of the expression in NP compared to the signal in notochord cells is also seen in the case of *Rgma*, *Rgmb*, and *Coro6*, while *Rgs7*, *Rgs11*, *Coro7* and in particular *Coro1a*, display the opposite trend, being detected at higher levels in NP than in notochord (Fig. 8A). Previous studies have shown that expression of notochord genes, including *Brachyury* and *Sonic hedgehog*, is higher in neonatal mouse NP cells, but decreases with age [32, 83–85]; IVD pathologies, including those in the NP cells, become evident around 2 years of age [83, 84, 86, 87]. Therefore, we validated the expression of select members from each gene family in NP cells microdissected from the lumbar discs of neonatal (one-week), middle-age (one-year), and very aged (two-year) wild-type mice in Friend leukemia virus B (FVB) background by multiplex qPCR analysis, using gene-specific TaqMan probes, and monitoring *Gapdh* expression as a control. We observed a significant increase in expression of *Phip* from one-week to one-year old mice (Fig. 8B). *Rgmb* expression also significantly increased from one-week to one-year old (Fig. 8C). On the other hand, *Tmod1* expression showed a significant decline from one-year to two-year old mice (Fig. 8D). We also tested the expression of *Rgs6*, however we did not detect this gene at any of the stages analyzed. *Coro7* expression was detected at all ages, with no significant changes in its expression (Fig. 8E).

**Fig. 9:**
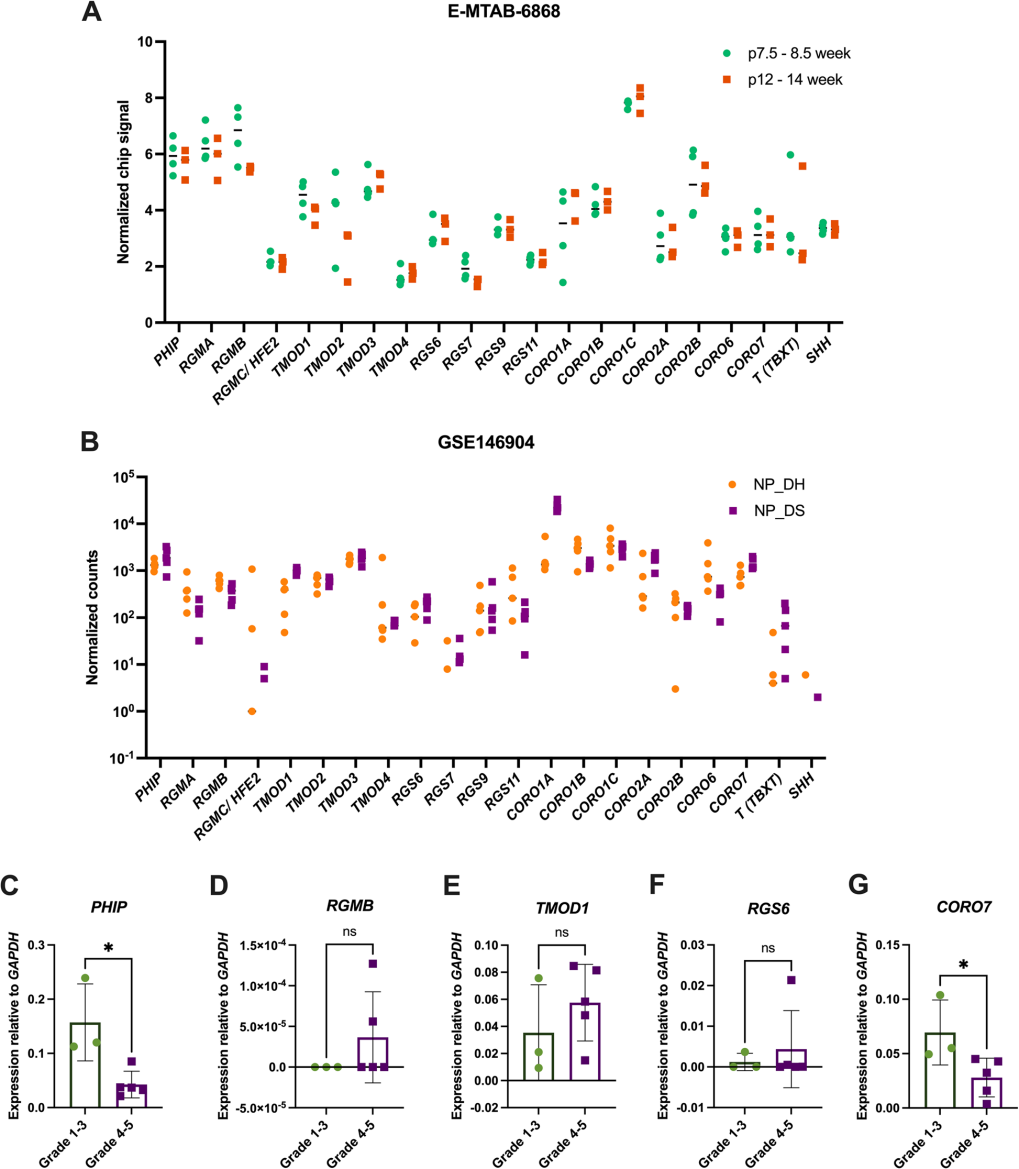
Expression of the genes of interest in human *nuclei pulposi*. **A** Normalized chip signals plotted as gene expression measures from microarray data (E-MTAB-6868; [88]) obtained from notochordal cells from human embryonic (7.5–8.5 weeks post-conception, *n* = 3) and fetal (12–14 weeks post-conception, *n* = 2) stages. **B** Log2 normalized read counts plotted as gene expression measures from the RNA-seq data of NP samples from lumbar disc herniation (DH, *n* = 5), and lumbar disc spondylolisthesis (DS, *n* = 5) obtained from the GEO database (GSE146904; [89]). Expression of PHIP (**C**), RGMB (**D**), TMOD1 (**E**), RGS6 (**F**), and CORO7 (**G**) relative to GAPDH, measured by qPCR analysis in the NP tissue collected from less degenerated (Grade 1–3) or moderately to severely degenerated (Grade 4–5) lumbar disc from human male and female of various age groups (see Methods). Results are presented as scatter dot plots with mean and standard deviation for each cohort. The qPCR results plotted in (C-G) were analyzed by unpaired *t* -test. * = *p* < 0.05. p, post-conception

To study the expression of the genes of interest in the human notochord and NP, we scanned the dataset obtained from microarray analyses of human notochord at 7.5, 8.5, 12 and 14 weeks post-conception (E-MTAB-6868; [88]). The normalized chip signals show that different members of the gene families analyzed here are detected at different levels in the human notochord (Fig. 9A). We next traced the expression of these genes using the bulk RNA-seq dataset from human NP cells collected from the lumbar discs of patients with two types of disc pathologies: disc herniation (DH) and degenerative spondylolisthesis (DS) (GSE146904; [89]). The normalized counts plotted in Fig. 9B show that the expression of most of the genes of interest is maintained in the postnatal NP cells of human IVDs. Of note, *RGMC/HJV/HFE2* (annotated as *RGMC/HFE2* in Fig. 9B) was expressed at very low levels.

Next, to validate the expression of our genes of interest during the progression of disc pathologies, we performed multiplex qPCR analysis on NP cells collected from human lumbar IVDs at early stage of IVD degeneration (Grade 1–3) and moderate to severe degeneration (Grade 4–5), using gene-specific TaqMan probes. The results indicate that expression of *PHIP* was significantly reduced with increased IVD pathologies severity (Fig. 9C). Expression of *RGMB, TMOD1,* and *RGS6* was detected in both cohorts, although the expression of *RGMB* was very low (Fig. 9D-F). As in the case of *PHIP*, the expression of *CORO7* showed a significant decline that correlated with the increased severity of IVD pathologies (Fig. 9G).

## Discussion

The notochord is a vital structure conserved throughout half a billion years of chordate evolution. During this time, the number of genes and the composition of the gene families expressed in the notochord and in other chordate hallmarks have been shaped by the two rounds of whole-genome duplication (WGD) seen in vertebrates, by an independent third WGD event specific to the teleost lineage, and by isolated lineage-specific gene duplication events, often counteracted by lineage-specific gene losses [64, 65, 90–92]. These events complicate the elucidation of the evolutionarily conserved complement of genes that confer to each structure its distinctive features. In this study, we sought to use the information on notochord genes that we had gained through a survey of notochord genes in *Ciona* to identify genes that could potentially be expressed in the notochord of different chordates and/or in the notochord remnants that compose the NP of murine and human IVDs. As a proof of principle, we have followed the evolutionary trajectories of five *Ciona* notochord genes in different vertebrates. We selected for this study one gene, *Phip*, which is not part of a multigenic family, and four genes that in vertebrates have become part of multigenic families. In *Ciona*, *Phip* is expressed in notochord, in mesenchymal cells, which are a population of small cells that give rise to most of the post-metamorphic tissues, and in the sensory vesicle, the region where the ocellus, a photoreceptive structure, and the otolith, a statocyst, are located. Interestingly, in addition to being expressed in the notochord, both *Danio* and *Xenopus phip* genes are detected in the developing eyes, and mouse *Phip* is reportedly expressed in the notochord, developing retina and inner ear [93]. Human *PHIP* is expressed in both notochord and NP.

The single-copy *RgmA/B/C/D* gene found in invertebrates has duplicated in vertebrates to give rise to four paralogs, *RgmA*, *RgmB*, *RgmC* and *RgmD*; however, only cartilaginous fish and teleosts seem to have the *RgmD* paralog [58], as confirmed by our synteny analysis. In *Ciona*, neural precursors are the earliest site of expression of *RgmA/B/C/D*, and it is only at the late tailbud stage that expression in notochord cells becomes detectable. On the other hand, expression in the sensory vesicle remains strong during early embryogenesis, and widens as the embryos progress through the tailbud stages. Interestingly, in addition to the expression in neurons during CNS development, notochord expression had been reported for three of the four zebrafish *rgm* genes, and the present study indicates that also the fourth zebrafish *rgm* gene, *rgmD*, is expressed in this structure. Similarly, *Xenopus rgmb* is strongly expressed in notochord, and also in the optic and otic vesicles; hence, the expression patterns of these genes in these vertebrates recapitulate the expression of the single-copy *Ciona* gene. *Rgmb* is also expressed in mouse and human notochord and NP, indicating conservation of expression in pre- and post-natal notochord cells of mammals.

The *Tmod* gene family is limited to a single member in most invertebrates, and has expanded to four components in vertebrates. In amphioxus, which is considered more distant than *Ciona* from vertebrates [10], there is only one *Tmod* gene, expressed in striated muscle [94]. Hence, the presence of a *Tmod* and a *Lmod* gene in *Ciona* suggests that the *leiomodin* genes were formed from *tropomodulin* genes via a duplication that occurred in the common ancestor of Olfactores (tunicates and vertebrates) [95]. Together with the reported expression of both *Tmod* and *Lmod* genes in the notochord of *Xenopus* [79], our results suggest that the notochord expression might have been an ancestral feature shared by *Tmod* and *Lmod* genes, which has been lost by *Ciona Lmod*. To study this gene family in vertebrates, we analyzed *Tmod2* paralogs from *Danio* and *Xenopus*, and found that both are expressed in notochord cells. When we tested expression of *Tmod1* in mouse and human NP, we found that also in these mammals *Tmod1* orthologs are expressed in these structures at high levels.

*Rgs6*, *Rgs7*, *Rgs9* and *Rgs11* are highly related to one another, and are predominantly expressed in neurons [39]. In particular, Rgs6 is reportedly the only member of the Rgs family able to inhibit the function of different receptors involved in neurotransmission, and for this reason its deletion is associated with phenotypes ranging from problems with the parasympathetic regulation of the heart rate to neuropsychiatric disorders [96–98]; loss-of-function mutations in this gene can also lead to cancer growth, due to the inactivation of its tumor-suppressing ability [99]. Studies carried out in human mesenchymal cells indicate that RGS5, RGS7 and RGS10 promote chondrogenesis, while RGS4 inhibits this process [100]. Our phylogenetic reconstruction suggests that *Ciona Rgs6/7* is more closely related to vertebrate *Rgs6* and *Rgs7* compared to *Rgs6/7* genes from amphioxus and non-chordate invertebrates. Together with the role of RGS7 in chondrogenesis and the evolutionary and histological relationships between notochord and cartilage [101], these findings suggest that *Ciona Rgs6/7* might contribute to notochord formation. Through this comparative study we elucidated the expression of *Xenopus Rgs6* in notochord, brain, and otic vesicles, which is reminiscent of the expression of *Ciona Rgs6/7*. Although published bulk RNA-Seq and microarray data suggest that *Rgs6* orthologs are expressed in the mouse and human notochord, respectively, we were able to validate expression of *RGS6* in human NP but not in mouse NP, which might be indicative of a difference in the molecular composition of the NP during the postnatal stages of these two species.

Coronins have been associated with a plethora of cellular processes, including auto-immunity, neuronal development and cancer progression [43]; however, a direct involvement of any member of this large family in notochord formation is yet to be reported. *Coronin 1A*, one of the best-characterized members of this family, is reportedly expressed in osteoclasts, where it functions as a regulator of bone resorption [102]. Recent studies have determined that in both *Drosophila* and mammalian cells, Coro7 interacts with core components of the Hippo pathway, and is required for its activation in response to various stimuli, including cell–cell contact [103]. We found that the closely related *Coro7* genes of *Danio* and *Xenopus* are both expressed in notochord cells, akin to the *Ciona* gene, and that their mouse and human counterparts are expressed in both notochord and postnatal NP.

Except for the case of *Ciona Rgs6/7*, which is downregulated in the primary notochord during the tailbud stages, we did not detect differences in gene expression along the rostro-caudal axis of the notochord in any of the species analyzed here. Regional differences in the expression of notochord genes appeared early in the chordate evolutionary timeline, having been reported in both tunicates [104, 105] and cephalochordates [106, 107], and they have been proposed to regulate regional morphogenesis in the mouse notochord [108]. Since we relied on RNA-Seq data in the case of mouse and human embryos, it remains to be determined whether any of the genes from the current study displays regional differences in its expression in developing mammals.

Interestingly, our analysis of differential gene expression in mouse NP uncovered a significant increase in the expression of *Phip* and *Rgmb* associated with aging. Since Phip proteins are modulators of the insulin pathway [33, 34], and Rgm proteins are co-receptors and regulators of the BMP signaling pathway [109], these results could be interpreted as part of a compensatory mechanism aimed at counteracting NP senescence. On the other hand, the decline of *Tmod1* expression could represent a read-out of the aging process. We also found that in human NP clinical samples, *PHIP* and *CORO7* expression significantly declined as NP degeneration increased; while the reasons and consequences of these changes in gene expression remain to be addressed, these findings open the possibility that these genes could become novel candidate diagnostic markers of human NP degeneration.

In conclusion, the results of this study add new candidate components to the notochord genetic toolkit shared by divergent chordates, and have uncovered changes in the expression of some of these genes that might be associated either with the transition from notochord to NP or with NP aging and degeneration.

## Materials and methods

### *Ciona robusta* embryo culture and whole-mount in situ hybridization (WMISH)

Adult *Ciona robusta* were purchased from Marine Research and Educational Products (M-REP; Carlsbad, CA) and kept at 16 °C in recirculating artificial seawater. Embryo cultures, fixation and staining were carried out as previously described [104]. Embryos were staged following the developmental timeline established in Hotta et al. [110]. Digoxigenin-labeled antisense RNA probes were synthesized in vitro using as templates *Ciona robusta* EST clones [111–113] (Table S1) linearized through appropriate restriction enzymes. WMISH experiments were performed as previously described [46, 104], using a hybridization temperature of 42 °C. After signal detection was satisfactorily completed (~ 4–48 h.), embryos were rinsed in 100% ethanol, washed briefly in xylenes, mounted in Permount (ThermoFisher Scientific, Waltham, MA) and photographed using a Leica DMR microscope (Leica Microsystems Inc., Buffalo Grove, IL).

### Scanpy analysis of single-cell RNA-Seq datasets

We utilized the single-cell RNA sequencing (scRNA-Seq) dataset available for *Ciona robusta* developing embryos from gastrula to larva stages [50]. We performed this analysis on the normalized and log-transformed notochord gene expression matrix available in the Gene Expression Omnibus (GEO) under accession number GSE131155, using the Scanpy (Single-cell analysis in Python https://scanpy.readthedocs.io/en/stable/) toolkit for the visualization of single-cell gene expression data [114].

### Evolutionary analyses

The protein sequences used for the phylogenetic surveys were retrieved from the NCBI and Ensembl databases using *Ciona robusta* proteins as initial queries for tBLASTn searches of the genomes of the organisms included in Figs. 2, 3, 4 and 5 [115]; reciprocal BLAST searches were performed using the Aniseed/WashU *Ciona robusta* genome browser (https://www.aniseed.fr) [52]. The sequences selected for phylogenetic analyses and their corresponding accession numbers are listed in Supplementary files 1–5. Sequence orthology was initially assessed using the reciprocal best BLAST hit approach, utilizing default parameters, and was later corroborated by phylogenetic analyses. The protein sequences were aligned by ClustalW using default parameters [116]. Phylogenetic trees were computed with the Maximum Likelihood (ML) inferences using PhyML 3 [117], employing automatic Akaike Information Criterion (AIC) by Smart Model Substitution (SMS) [118], which selected the Jones-Taylor-Thornton (JTT) substitution model, 0.4 as the proportion of invariable sites (I) and 4 as the gamma distribution parameter (γ) [119]. Branch support was provided by aLRT (approximate likelihood ratio test) [120]. Domain analyses were carried out employing the PROSITE database [121] and InterPro software [122].

First-pass synteny analyses were carried out using the Genomicus genome browser [123] (https://www.genomicus.bio.ens.psl.eu/genomicus-109.01/cgi-bin/search.pl), using a window of twenty genes. The results were cross-referenced and detailed using species-specific UCSC (https://genome.ucsc.edu) and Ensembl (https://www.ensembl.org) genome browsers.

### Zebrafish handling, probe synthesis and WMISH

Zebrafish (*Danio rerio*) embryos were obtained from natural spawning of wild-type animals. The embryos were fixed overnight in 4% paraformaldehyde (PFA) in phosphate-buffered saline (PBS), washed three times in pre-chilled 1 × PBT (PBS/0.1% Tween), then three times in cold methanol, and stored in methanol at -20 °C until use. All the protocols for handling of zebrafish and experiments that involve non-feeding larvae were approved by the local review panel. The sequences of the zebrafish genes of interest were retrieved from the NCBI database using the corresponding *C. robusta* coding regions as queries for BLASTn searches [124]. ESTs were found only for *rgs7a* and *tmod2*; templates for RNA probe synthesis for the remaining genes were cloned using the oligos listed in Table S2. PCR-amplified gene fragments were cloned into the pGEM®-T Easy vector (Promega, Madison WI) and 500 ng of purified template DNA were used for in vitro transcription of digoxygenin-labeled RNA probes with SP6 and T7 RNA polymerases (Roche, Indianapolis, IN). All RNA probes were purified using 4 M lithium chloride and stored in formamide at -80 °C until use.

WMISH was carried out as previously described [74, 125]. In short, embryos were re-hydrated and permeabilized through digestion with Proteinase K (10 μg/ml), followed by five washes in PBT. After 1 h of post-fixation at RT in 4% PFA dissolved in PBS, embryos were rinsed with PBT four times and hybridized overnight at 65 °C in hybridization buffer [125]. After the hybridization solution was removed, embryos were washed several times in maleic acid buffer and incubated overnight at 4 °C with anti-digoxygenin-AP antibody (Roche, Indianapolis, IN). The staining reaction was performed at room temperature employing BM Purple (Roche, Indianapolis, IN). Images of stained embryos were captured using a Zeiss Axio Imager M1.

### *Xenopus laevis* handling, probe synthesis, WMISH and histology

*Xenopus laevis* embryos were staged according to Nieuwkoop and Faber [126] and raised in 0.1X NAM (Normal Amphibian Medium; [127]). All the procedures used for these experiments were approved by the New York University Institutional Animal Care and Use Committee (IACUC animal protocol #150,201).

*Xenopus laevis phip.S, coro7.S, tmod2.L, rgmb.S and rgs6.S* were amplified by PCR (S100 Thermal Cycler; Biorad, Hercules, CA) from NF stage 11.5 (*phip.S, coro7.S* and *rgmb.S)* or NF stage 25 (*tmod2.L* and *rgs6.S*) cDNA with the primer sets described in Table S3, using Illustra PuReTaq™ Ready-To-Go™ PCR Beads (GE Healthcare, Chicago, IL). The PCR conditions were as follows: denaturation at 95 °C (30 s), annealing at 60 °C (60 s) and extension at 72 °C (90 s) for 35 cycles. The PCR products recovered were cloned into pGEM®-T Easy (Promega, Madison, WI), sequenced, and linearized to generate sense and antisense in situ hybridization probes.

Embryos at the appropriate developmental stages (NF stage 28 and 32) were fixed in MEMFA (0.1 M 3-N-Morpholino-propanesulfonic acid pH 7.4, 2 mM EGTA, 1 mM MgSO4 and 3.7% formaldehyde), and processed for in situ hybridization. For each gene, sense and antisense digoxygenin-labeled probes (Genius kit; Roche, Indianapolis, IN) were synthesized using the corresponding linearized pGEM®-T Easy construct. WMISH was performed as described [128, 129]. For histology, NF stage 32 stained embryos were embedded in Paraplast + (Sigma-Aldrich, St. Louis MO), sectioned (12 µm) on a rotary microtome (Cut4060; Olympus, Center Valley, PA), counterstained with Eosin Y (Sigma-Aldrich, St. Louis MO) and mounted in Permount (ThermoFisher Scientific, Waltham, MA). Embryos and sections were imaged on a Leica M165 Stereomicroscope (Leica Microsystems Inc., Buffalo Grove, IL). Staining was confirmed on four different batches of embryos.

### Mouse *nucleus pulposus* cell collection

Wild-type female and male in FVB background mice used in these studies were maintained in a temperature-controlled facility with equal light–dark cycle and food and water provided ad libitum, in accordance with the National Institutes of Health Guide for the Care and Use of Laboratory Animals. All experiments were carried out in accordance with institutional guidelines under IACUC approved at Weill Cornell Medical College (WCMC) under the IACUC protocol number 2016–0026.

FVB mice at one week of age (*n* = 3), one-year (*n* = 6), and two-years (*n* = 6) of age were used to analyze the expression of *Phip, Rgmb, Tmod1, Rgs6,* and *Coro7* genes in NP cells. The NP cells were microdissected from the IVDs of lumbar spine in cold PBS underneath a Nikon bright-field stereomicroscope (Nikon, Japan) as we described recently [130]. The NP cells from each biological replicate were directly collected in RNA*later*™ (Invitrogen by Thermo Fisher Scientific, Lithuania, AM7024) and stored at 4 °C for 24 h.

### Human *nucleus pulposus* tissue collection

NP tissue was collected under the Hospital for Special Surgery (HSS) Institutional Review Board (IRB) approved research study and protocol number 2016–933, all in compliance with the applicable requirements of the FDA regulations and HSS regulations. Patients who were recruited for this study were undergoing spine surgery due to prior medical diagnosis and treatment. Informed consent was obtained to collect the NP tissue, which otherwise would have been discarded after the surgical procedure. The T2-weighted MRI image taken prior to surgery was used to assess the Pfirrmann grade of the disc. Pfirrmann grading [131] is the gold standard for the quantification of disc health based on water content. Grade 1–2 IVDs are considered healthy; Grade 3 IVDs show early sign of disc degeneration and Grade 4–5 IVDs are moderately to severely degenerated. A total of eight samples were analyzed in the current study from males and females between 19–79 years of age. In the present study, we combined NP samples from Grade 1–3 (*n* = 3) in one cohort, and compared them to NP samples from Grade 4–5 (*n* = 5). Following surgery, the samples were immediately stored on ice and delivered to the lab, where they were weighed, washed three times in PBS, and stored in RNA*later*™ (Invitrogen by Thermo Fisher Scientific, Lithuania, AM7024) at 4 °C for 24 h.

### Mouse and human *nucleus pulposus* RNA isolation and qPCR analysis

Total RNA was isolated by extraction in TRI-reagent (Sigma-Aldrich, St. Louis MO), followed by purification and elution using Qiagen RNeasy Kit (Qiagen, Germany; cat. #74,004 for mouse, and cat. #74,707 for human cells), as previously described [130]. RNA concentration was quantified in duplicates using a NanoDrop™ One Microvolume UV–Vis Spectrophotometer (Thermo Scientific, USA, AZY1601393). The RNA was immediately converted into cDNA using the SuperScript™ IV First-Strand Synthesis System (Invitrogen by Thermo Fisher Scientific, Lithuania, 18,091,050). Multiplex qPCR was performed using the CFX96 Touch™ Real-Time PCR Detection System (Bio-Rad, Singapore, 1,855,195). Each reaction used 8 ng of cDNA, iQ™ Multiplex Powermix (Bio-Rad, USA, 1,725,849) master mix, gene- and species-specific TaqMan™ probes (Table S4) conjugated to FAM-MGB (Thermo Scientific, USA), and an internal control (*Gapdh*) conjugated to VIC-MGB (Thermo Scientific, USA). The data are presented as median and relative to the reference gene. The Cq values obtained for each gene were subtracted from the Cq values of *Gapdh* to obtain the delta Cq values. The logarithm of the -deltaCq value was calculated to plot the expression of each gene relative to *Gapdh*. The qPCR results in mouse NP cells compared three age points (one week, one and two year; see above) and were analyzed by ordinary one-way ANOVA followed by Tukey’s multiple comparisons test to determine statistical differences in gene expression among different age groups. The qPCR results from human NP cells collected from Grade 1–3 and Grade 4–5 were analyzed using unpaired *t-*test.

### Analysis of gene expression using existing mouse and human datasets

Single-cell data were obtained as SingleCellExperiment objects from the Bioconductor package [132]. The raw sequencing data in the MouseGastrulationData package were acquired from ArrayExpress (accession E-MTAB-6967), analyzed using Seurat [133] and processed as described in Pijuan-Sala et al., 2019 [81].

RNA sequencing (RNA-seq) data from mouse notochord cells at E12.5 and NP cells at P0 were obtained from the Gene Expression Omnibus (GEO) database (GSE100934) [82]; read counts were normalized using the DESeq2 method [134], and the normalized values are plotted as gene expression measures. Microarray data (E-MTAB-6868; [88]) obtained from notochordal cells from human embryonic (7.5–8.5 week, *n* = 3) and fetal (12–14 weeks, *n* = 2) stages post-conception were downloaded and the intensity values were extracted and normalized. The normalized gene expression values were plotted as gene expression measures. RNA-seq data from 10 human NP samples of lumbar degenerated discs (DH, herniated and DS, spondylolisthesis; *n* = 5 for each tissue) were obtained from the GEO database (GSE146904; [89]), and Log2 normalized read counts were plotted as gene expression measure.

### Supplementary Information

**Additional file 1: Figure S1.** Alignment of Phip protein sequences.

**Additional file 2: Figure S2.** Comparative view of the genomic context of the single-copy *RgmA/B/C/D* genes found in hemichordates and tunicates and of *RgmB* genes of vertebrates.

**Additional file 3: Figure S3.** Comparative view of the genomic context of vertebrate *RgmA* and *RgmC* genes.

**Additional file 4: Figure S4. **Comparative view of the genomic context of the fish-specific *RgmD* genes.

**Additional file 5: Figure S5.** Comparative view of the genomic context of vertebrate *Tmod2* and *Tmod3* genes.

**Additional file 6: Figure S6.** Comparative view of the genomic context of hemichordate, tunicate and vertebrate *Coro7* genes.

**Additional file 7: Figure S7.** Expression of members of the gene families analyzed in this study during select stages of mouse gastrulation.

**Additional file 8: Table S1.** *Ciona robusta* gene models and ESTs.

**Additional file 9: Table S2.** *Danio rerio* gene models, ESTs and cloning primers.

**Additional file 10: Table S3.** *Xenopus laevis* gene models and cloning primers.

**Additional file 11: Table S4.** Mouse and human gene models and probe IDs.

**Additional file 12: Table S5.** ENSEMBL gene models for the mouse genes in Fig. S7.

**Additional file 13: Supplemental file 1.** Phip protein sequences used in Fig. S1, with their respective accession numbers.

**Additional file 14: Supplemental file 2.** Rgm protein sequences used in Fig. 2, with their respective accession numbers.

**Additional file 15: Supplemental file 3.** Tmod protein sequences used in Fig. 3, with their respective accession numbers.

**Additional file 16: Supplemental file 4.** Rgs protein sequences used in Fig. 4, with their respective accession numbers.

**Additional file 17: Supplemental file 5.** Coronin protein sequences used in Fig. 5, with their respective accession numbers.

## Data Availability

All data generated during this study are included in this published article and its supplementary information files. Data sharing is not applicable to this article as no datasets were generated during the current study.

## Abbreviations

aLRT: Approximate likelihood ratio test
ANOVA: Analysis of variation
cDNA: Complementary DNA
CNS: Central nervous system
EST(s): Expressed Sequence Tag(s)
FVB: Friend leukemia virus B
hpf: Hours post fertilization
hr(s): Hour(s)
kb: Kilobase(s), or 1000 base pairs
ML: Maximum likelihood
NAM: Normal Amphibian Medium
NF: Nieuwkoop Faber
NP: *nucleus pulposus/nuclei pulposi*
nt: Nucleotide(s)
ORF: Open reading frame
(q)PCR: (Quantitative) Polymerase Chain Reaction
P0: Post-natal day zero
PFA: Paraformaldehyde
PBS: Phosphate-buffered saline
PBT: PBS/0.1% Tween
WGD: Whole-genome duplication
WMISH: Whole-mount in situ hybridization
