# Supplementary material for: Gene expression in notochord and nuclei pulposi: a study of gene families across the chordate phylum
Source: BMC Ecol Evol. 2023 Oct 27;23:63. doi: 10.1186/s12862-023-02167-1 (PMC10605842; doi:10.1186/s12862-023-02167-1)
Supplement: Supplementary file 1 — Additional file 1: Figure S1. Alignment of Phip protein sequences. [file 12862_2023_2167_MOESM1_ESM.pdf]

Figure S1. Alignment of Phip protein sequences.

Alignment of Phip proteins from the species analyzed in this study and other representative metazoans, showing the degree of conservation of the five WD repeats (turquoise), and the two bromodomains (green). The species analyzed in this study are in bold font.

```
Phip Nematostella vectensis             ----------------------------------------ELYFLVAKFLSGGPCKRAAE         20
Phip Strongylocentrotus purpuratus      ------------------------MAEKDTGRINDSTTESELYYLIAKFLESGPCKESAN         36
Phip Branchiostoma belcheri             ------------------------METTGSTSREASPTESELYFLIAKFLSGGPCTRAFE         36
Phip Ciona robusta                      RYCRVFKIIQPSSLLFLSISRCRLCCNSIGRKMESKNLNLDLYFLIERFLSKGPCAPVSK         60
Phip Petromyzon marinus                 -----------------------MAARPSHGGGGVSRLESELFFLIARFLSAGPCREAAE         37
Phip Lepisosteus oculatus               ---------------------------MASDSKHVAQLKSELYFLIARFLEAGPCQNAAE         33
Phip Danio rerio                        ---------------------------MAAERKHVAQLKSELYFLIARFLEAGPCQDAAE         33
Phip Latimeria chalumnae                ---------------------------MSCDSKHIKELKSELYFLIARFLEDGPCQQAAE         33
Phip Xenopus tropicalis                 --------------------------MSSLNNQHSTELKSELYFLIARFLEDGPCQQAAQ         34
Phip Gallus gallus                      -------------------------MSGDGGSRHTAELRAELYFLIARFLEDGPCQQAAQ         35
Phip Mus musculus                       ---------------------------MSRERKGLSELRSELYFLIARFLEDGPCQQAAQ         33
Phip Homo sapiens                       ---------------------------MSCERKGLSELRSELYFLIARFLEDGPCQQAAQ         33
                                                                                :*::*: :**. ***    :

Phip Nematostella vectensis             ILMHAMFTRHDLLPISQQLLPRRLDWEGNSHPKSYQET----SYENRHISVDFLAQISSR         76
Phip Strongylocentrotus purpuratus      VLRRQIE--------QNGLFSARFDWKGNKHQQSLKNL----DNCNGHIKNDHLLKIVGR         84
Phip Branchiostoma belcheri             VLQQELQ--------EHELLERRSDWLGNTHPQTFGNL----VQRNPHVSQQHLLQVCKR         84
Phip Ciona robusta                      VLREVLN--------SLNIVPKRFDWKGGQHVQSYDEW----TRRFSDVGDDHLLVLCNH         108
Phip Petromyzon marinus                 VLVREIE--------QHKLLPRRVDWRGAEHERSFAEM----VLSHTHLVPEHLLLVVQR         85
Phip Lepisosteus oculatus               TLIKEVE--------EKELLPKRTDWTGKEHPGTYENL----VKIYRHVAPDHLLQICRC         81
Phip Danio rerio                        TLIREVQ--------QKELLPKRIDWTGKEHPGSYENL----VKLYRHISPDHLLQVCER         81
Phip Latimeria chalumnae                VLIREVE--------EKQLLPKRTDWMGKEHPRTYENLLMQKVKCYRHIAPDHLLRICQR         85
Phip Xenopus tropicalis                 VLIREVE--------EKELLPKRVDWTGKEHARTYQNL----VKYYRHLAPDHLLQICQR         82
Phip Gallus gallus                      VLIREVA--------EKELLPKRTDWTGKEHPRSYQNL----VKYYRHLAPDHLLQICHR         83
Phip Mus musculus                       VLIREVA--------EKELLPRRTDWTGKEHPRTYQNL----VKYYRHLAPDHLLQICHR         81
Phip Homo sapiens                       VLIREVA--------EKELLPRRTDWTGKEHPRTYQNL----VKYYRHLAPDHLLQICHR         81
                                         * . :         .  :.  * ** *  *  :  :          .:  :.*  :

Phip Nematostella vectensis             LCPLLDKCVPSSVRGVQTLLGAGRQSLLRTGSGFIHPT--VSSLALCNHGA--PLLPPIG         132
Phip Strongylocentrotus purpuratus      IGHLLDKDTRPRVPGVKSLLGGARQSLLRTVADVKPPLWSPSVHAALYHHR--PIAPPNK         142
Phip Branchiostoma belcheri             VGPLLDAQVRPSVPGVRTLLGLGRQSLLRHADDFKKVQWKGPTLAAKASGSHGNLMPPVN         144
Phip Ciona robusta                      ITSMLDQKS--LNRGETSLLQVGRNSLSSD----EDIVWKFCHTVANINAK--PMIPPHN         160
Phip Petromyzon marinus                 LGPLLDREIPPSVPGVCSLLGAGRQSLLRTRKDLGRVVWRGSALAALHRGR--PPQPQSS         143
Phip Lepisosteus oculatus               IGPLLEKEVPASVPGVQSLLGAGRQSLLRTNKSCKHVVWKGSALAALHCGR--PPEPPVN         139
Phip Danio rerio                        VSPLLEREVPASVPGVNSLLGTGRQSVLRTNKSCKHVVWKGSALAALHCGR--PPEPPVI         139
Phip Latimeria chalumnae                VEPLLEREIPPSVPGVQSLLGAGRQSLLRTNKSCKHVIWKGSTLAALHCGR--PPEPPIN         143
Phip Xenopus tropicalis                 IGPLLEKEVPQSVPGVQTLLGAGRQSLLRTNKSCKHVVWKGSALAALHCGR--PPEPPIN         140
Phip Gallus gallus                      LGPLLEQEIPQSVPGVQTLLGAGRQSLLRTNKSCKHVVWKGSALAALHCGR--PPESPVN         141
Phip Mus musculus                       LGPLLEQEIPQSVPGVQTLLGAGRQSLLRTNKSCKHVVWKGSALAALHCGR--PPESPVN         139
Phip Homo sapiens                       LGPLLEQEIPQSVPGVQTLLGAGRQSLLRTNKSCKHVVWKGSALAALHCGR--PPESPVN         139
                                        :  :*:        *  :**  .*:*:                 .

Phip Nematostella vectensis             KRLRPNIAQVLLGRELSGPAVKRQLAQP-NVYVKQSMHRRILGHLSAVYCVLFDRTGQCI         191
Phip Strongylocentrotus purpuratus      LSQVPNVYNVIRGRQLAGVARLDHIIPG-ERYSRMNLHLRSLSHLSSAYCVLFDQTGRRI         201
Phip Branchiostoma belcheri             YGHAPAACHVVNARQLLGVAKRHFLAPAQTTYSKYSMHLRVLGHLSAVYCIAFDRTGKCI         204
Phip Ciona robusta                      YRKKQNWIHSIKSREYGCGRLKQVVCPVVPVYGKMNMCLRVLGHLSSVYCVLFDRTGSKI         220
Phip Petromyzon marinus                 VGNPPHLVSLLSARERTGCIRHGHAVPI-TLYQHARMHRRILGHLSSVYCLAFDRTGQRI         202
Phip Lepisosteus oculatus               YGSPPNIVETAYGRRLNGTYRLGQLVPT-AVYQHMKMHKRILGHLSSVYCVTFDRTGRRI         198
Phip Danio rerio                        FGSPPNIAETIHSRRLNGSYRLGQLVPT-AVYQHMKMHKRILGHLSSVYCVTFDRTGRRI         198
Phip Latimeria chalumnae                YGRPPNIVDTTFSRCLNGTYRLGQLVPT-AVYQHMKMHKRILGHLSSVYCVTFDRTGRRI         202
Phip Xenopus tropicalis                 YGSPPSIANTFFARKLNGNYQFENLVPT-AVYQHMKMHKRILGHLSSVYCVTFDRTGRRI         199
Phip Gallus gallus                      YGSPPSIVDTLFSRKLNGKYRLERLVPT-AVYQHMKMHKRILGHLSSVYCVTFDRTGRRI         200
Phip Mus musculus                       YGSPPSIADTLFSRKLNGKYRLERLVPT-AVYQHMKMHKRILGHLSSVYCVTFDRTGRRI         198
Phip Homo sapiens                       YGSPPSIADTLFSRKLNGKYRLERLVPT-AVYQHMKMHKRILGHLSSVYCVTFDRTGRRI         198
                                                    .*                 * :  :  * *.***:.**: **:**  *

Phip Nematostella vectensis             ITGADDSLVKIWSTKDGRLLATLRGHNGEVSDIAINYENTLVAAGSCDKLIRVWSLQTTY         251
Phip Strongylocentrotus purpuratus      FTGADDHLVKIWSVDDGRLLATLRGHAAEIVDMNVNYENTLLAAASNDKVIRVWNLQSLT         261
Phip Branchiostoma belcheri             ITGADDNLLKIWSAHDGRLVATLRGMSSEITDMAVNYENTLLAAGSVDKTIRVWDMKTLA         264
Phip Ciona robusta                      FTGSDDSLIKCWCAWSGRLLFTFRGHQAEICDMDISYDNILLAAGSCNKEIRVWDVQTAE         280
Phip Petromyzon marinus                 FTGSDDCLVKVWSAEDGRLLATLRGHAAEISDMAVNYENSLLAAGTCDHVVRVWCLRTCA         262
Phip Lepisosteus oculatus               FTGSDDCLVKIWATDDGRLLATLRGHAAEISDMAVNYENTMIAAGSCDKTIRVWCLQTCA         258
Phip Danio rerio                        FTGSDDCLVKIWATDDGHLLATLRGHAAEISDMAVNYENTLLAAGSCDKMIRVWCLQTCA         258
Phip Latimeria chalumnae                FTGSDDCLVKLWTTDNGRLLATLRGHAAEISDMAVNYENTMIAAGSCDKMIRVWCLRTCA         262
Phip Xenopus tropicalis                 FTGSDDCLVKIWSTDDGRLLATLRGHAAEISDMAVNFENTMIAAGSCDKMIRIWCLRTCA         259
Phip Gallus gallus                      FTGSDDCLVKIWATDDGRLLATLRGHAAEISDMAVNYENTMIAAGSCDKMIRVWCLRTCA         260
Phip Mus musculus                       FTGSDDCLVKIWATDDGRLLATLRGHAAEISDMAVNYENTMIAAGSCDKMIRVWCLRTCA         258
Phip Homo sapiens                       FTGSDDCLVKIWATDDGRLLATLRGHAAEISDMAVNYENTMIAAGSCDKMIRVWCLRTCA         258
                                        :**:** *:* * . .*:*: *:**  .*: *: :.::* ::**.: :: :*:* :::

Phip Nematostella vectensis             PVAVLQGHTGMITSLQFCPSPASDARYLMSTGGDGTVCFWKFNPANYLFDPKPLRFIEKS         311
Phip Strongylocentrotus purpuratus      PVAVLMGHTAMITSLEFSPYVRGSARYMASTGADGCVCIWVWEKETLKFNNKPIKFQERN         321
Phip Branchiostoma belcheri             PVSVLMGHSAMVTSLQFCPSARGSQRFLASTGADGTVCFWQWCVNTCTFNQTPIKFADRS         324
Phip Ciona robusta                      PVAVLQGHSGILTSLEFSPVVECGRGYLISTGRDGNVCFWLWNINNMEFNKQPVKFQERS         340
Phip Petromyzon marinus                 PMAVLQGHTASITSLQFSPQCRGSVRWLASSGADGIVCFWQWDAASLKFCERPVKFTERT         322
Phip Lepisosteus oculatus               PLAVLEGHGALITSLQFSPLCSGSKRYLSSTGADGSICFWQWDARTLKFSQRPSKFTERP         318
Phip Danio rerio                        PLAVLEGHAASITSLQFSPLCSGNKRFLSSTGADGTICFWQWDARTLKFGQRPSKFIERP         318
Phip Latimeria chalumnae                PIAVLQGHSASITSLQFSPLCSGSKRFLSSTGADGIICFWQWDARTLKFSHRPSKFTERP         322
Phip Xenopus tropicalis                 PLAILQGHSASITSLQFSPLCNGSKRYLSSTGADGTICFWLWDAGTLKINPRPTKYTERP         319
Phip Gallus gallus                      PLAVLQGHSASITSLQFSPLCSGSKRYLSSTGADGTICFWLWDAGTLKINPRPTKFTERP         320
Phip Mus musculus                       PLAVLQGHSASITSLQFSPLCSGSKRYLSSTGADGTICFWLWDAGTLKINPRPTKFTERP         318
Phip Homo sapiens                       PLAVLQGHSASITSLQFSPLCSGSKRYLSSTGADGTICFWLWDAGTLKINPRPAKFTERP         318
                                        *:::* ** . :***:*.*    .  :: *:* ** :*:* :   .  :   * :: ::

Phip Nematostella vectensis             RPGTQMVCSSFSPGGSFLVAGSTDHIIRIYQMSPGPPE--RIAELDVHSDHVDSIQFNHT         369
Phip Strongylocentrotus purpuratus      RPGSQMLCLSFSPGGTFLVAGGTDTAIRVYNFNTIPPE--KVTELEAHTDRVDSIGFCHT         379
Phip Branchiostoma belcheri             RAGAQLLCSSFSPGGTFLVTGNSDHIIRVYFLSVAGPD--RIAELEAHTDKVDSIQYNNS         382
Phip Ciona robusta                      RPGVGIMCASWSMGGRFAVAGGSDSIIHVYCISPGSPEPLKLCELSSHSDKVDSIWFSHS         400
Phip Petromyzon marinus                 RPGVQIFCSSFSAGGMFLATGSTDHVIRVYYLGDRSPD--KIAELEAHTDKVDSIQFSNN         380
Phip Lepisosteus oculatus               RPGVQMICSSFSAGGMFLATGSTDHIIRVYFFGSGQPE--KISELESHTDKVDSIQFSNT         376
Phip Danio rerio                        RPGVQMICSSFSAGGMFLATGSTDHIIRVYYFGGGQPE--KISELESHTDKVDSIQFSHS         376
Phip Latimeria chalumnae                RPGVQMICSSFSAGGMFLATGSTDHIIRVYFFGSGQPE--KISELECHTDKVDSIQFCNT         380
Phip Xenopus tropicalis                 RPGVQMICSSFSSGGMFLATGSTDHVIRVYYFGSGQPE--KISELEFHTDKVDSIQFSNT         377
Phip Gallus gallus                      RPGVQMICSSFSAGGMFLATGSTDHIIRVYFFGSGQPE--KISELEFHTDKVDSIQFSNS         378
Phip Mus musculus                       RPGVQMICSSFSAGGMFLATGSTDHIIRVYFFGSGQPE--KISELEFHTDKVDSIQFSNT         376
```

```
Phip Homo sapiens                   RPGVQMICSSFSAGGMFLATGSTDHIIRVYFFGSGQPE--KISELEFHTDKVDSIQFSNT   376
                                    * *  :.* *:* ** * .:*.:*  *::* :.   *:  :: **. *:*:**** : :.

Phip Nematostella vectensis         GEKFLSGSRDGTARIWFFKRSKWEHILLDITRTLPGGQETQ-VVADKCLNCHMFQVAWDL   428
Phip Strongylocentrotus purpuratus  GSRFISGSKDGTARVWRYERQGWKNILLNMNSRLPGSKAPKLDEGNKIVKHRVTMVGWNK   439
Phip Branchiostoma belcheri         GDKFVSGSRDGTARVWRFERQEWRNVLLNMATSLPGKSNQL-DTANKYFKWKVTMVGWDL   441
Phip Ciona robusta                  GLQFASASCDGRAIIWSYVEAEWRSLTLNTDRRRNLNDD---GEFVSMSKVRVIMVTWTC   457
Phip Petromyzon marinus             GDRFVSGSRDGTARIWQYRRQEWKSLLLDMSSKLPG-NNNP-PVEDKVFKLKVTMVAWDR   438
Phip Lepisosteus oculatus           GDRFVSGSRDGTARIWQLHQQEWKSILLDMATKLSG-NNPP-PVEDKVNKLKVTMVAWDR   434
Phip Danio rerio                    GDRFVSGSRDGTARIWQLQQQDWRSILLDMATKLPGKYNPP-PLEDKVTKLKVTMVAWDC   435
Phip Latimeria chalumnae            GNRFVSGSRDGTARIWQFKRQEWRSILLDMATRPSG-HNPQ-GLEDKITKLKVTMVAWDR   438
Phip Xenopus tropicalis             GNRFVSGSRDGTARIWQFKRREWKSILLDMTTRPTG---VQ-GVEDKITKLKVTMVAWDR   433
Phip Gallus gallus                  SSRFVSGSRDGTARIWQFKRREWKSILLDMATRPSG-QTLQ-GVEDKITKLKVTMVAWDR   436
Phip Mus musculus                   SNRFVSGSRDGTARIWQFKRREWKSILLDMATRPAG-QNLQ-GIEDKITKMKVTMVAWDR   434
Phip Homo sapiens                   SNRFVSGSRDGTARIWQFKRREWKSILLDMATRPAG-QNLQ-GIEDKITKMKVTMVAWDR   434
                                    . :* *.* ** * :*   .  *. : *:                 .  : ::  * *

Phip Nematostella vectensis         WDRHVVTAINDHTLKVWEAETGALIHVLQGHHDEMFVLEAHPTDPHIFLSAGHDGLVILW   488
Phip Strongylocentrotus purpuratus  NDYYVVTAVNDHSLRVWNSYNGKLVYELYGHTDEVYVLEGHPKDVRIFLSGGHDGLIFLW   499
Phip Branchiostoma belcheri         HDKYVITAVNDKSLKVWDSHSGKLVHILEGHTDEVFVLEAHPTDPHVLLSAGHDGHVMVW   501
Phip Ciona robusta                  DDQHIMTSHSDFSIKVWKVDTASLLHVLKGHEDEAYCLEAHPNDGRILLSAGHDGKIFLW   517
Phip Petromyzon marinus             HDSTVITAVNTLALKVWSSYTGQLLHVLTGHEDEVFVLESHPVDPRILLSAGHDGNVIMW   498
Phip Lepisosteus oculatus           HDNTVITAVNNLTLKVWNSYTGQLIHVLMGHEDEVFVLEPHPFDPRVLFSAGHDGNAIVW   494
Phip Danio rerio                    NDNTVITAANNLTLKVWNSYTGNLIHVLMGHEDEVFVLEPHPFDSRVLFSAGHDGNAIVW   495
Phip Xenopus tropicalis             HDNFVITAVNNMTLKVWNSSNGQLVHILMGHEDEVFVLESHPFDPRVLFSAGHDGNVIVW   493
Phip Latimeria chalumnae            HDNTVITAVNNLTLKVWNSYTGQLIHVLMGHEDEVFVLEPHPFDPRVLFSAGHDGNVIVW   498
Phip Gallus gallus                  HDNSVITAVNNMTLKVWNSFTGQLIHILMGHEDEVFVLEPHPFDPRVLFSAGHDGNVIVW   496
Phip Mus musculus                   HDNTVITAVNNMTLKVWNSYTGQLIHVLMGHEDEVFVLEPHPFDPRVLFSAGHDGNVIVW   494
Phip Homo sapiens                   HDNTVITAVNNMTLKVWNSYTGQLIHVLMGHEDEVFVLEPHPFDPRVLFSAGHDGNVIVW   494
                                     *  ::*: .  :::**.  .. *:: * ** ** : ** ** * ::::*.****  ::*

Phip Nematostella vectensis         DLRTGQMIKSFFNSIEGQGHGAVFDCKFSPDGLQFACTDSHGHLCIFGYGSSDPFNKVPS   548
Phip Strongylocentrotus purpuratus  DLSTGQQLYRFLNTIEGQGFGSIYDCKFSPTGEQFVATDSHGHFSIFGMGSSKPYDKVPK   559
Phip Branchiostoma belcheri         DLQTGSLIKRFFNMIEGQGHGAVFDCKFSPTGMHFAATDSHGHLLIFGFGSKEPYSKIPE   561
Phip Ciona robusta                  DINDGQQVINFHNLLVGQGHGAVFDAKFSPTGNHIACTDSHGHLILFGFGDGKVYKKVPD   577
Phip Petromyzon marinus             DLSRGAKIRTYFNMIEGQGHGAVFDCKFSPDGQHFACTDSHGHLLIFGFGSSSKYDKIPD   558
Phip Lepisosteus oculatus           DLVRGVKIRSYFNMIEGQGHGAVFDCKCSPDGQHFACTDSHGHLLIFGFGSSSKYDKIAD   554
Phip Danio rerio                    DLARGVKIRSYFNMIEGQGHGAVFDCKCSPDGQHFACTDSHGHLLIFGFGSSSRYDKIAD   555
Phip Latimeria chalumnae            DLARGTKIRSYFNMIEGQGHGAVFDCKCAPDGQHFACTDSHGHLLIFGFGSSSRFDKIAD   558
Phip Xenopus tropicalis             DLARGVKIRSYFNMIEGQGHGAVFDCKCSPDGQHFACTDSHGHLLIFGFGSSSKYDKIAD   553
Phip Gallus gallus                  DLARGVKIRSYFNMIEGQGHGAVFDCKCSPDGQHFACTDSHGHLLIFGFGSSSKYDKIAD   556
Phip Mus musculus                   DLARGVKVRSYFNMIEGQGHGAVFDCKCSPDGQHFACTDSHGHLLIFGFGSSSKYDKIAD   554
Phip Homo sapiens                   DLARGVKIRSYFNMIEGQGHGAVFDCKCSPDGQHFACTDSHGHLLIFGFGSSSKYDKIAD   554
                                    *:  *  :  : * : ***.*:::*.* :* * ::..******: :** *. .  :.*: .

Phip Nematostella vectensis         EQFFHTDYRPLIRDANSYVLDEQTQTAPHLMPPPFLVDVDGNPHPSHVQRLVPGHGSNVR   608
Phip Strongylocentrotus purpuratus  EQFFHTDYRPLVRDANNYVLDEQTQQAPHLMPPPFLVDIDGNPHPPRYQRLVPGRENCKD   619
Phip Branchiostoma belcheri         EMFFHTDYRPLIRDSNNYVLDEQTQQAPHLMPPPFLVDVDGNPHPPSLQRLVPGRENMPA   621
Phip Ciona robusta                  EQFFHTDYRPLLRDMYGAVVDEQTHQAPNRMPPPFLVDVDGNPHPAPLQRLVPGRQNITT   637
Phip Petromyzon marinus             QMFFHTDYRPLLRDANNFVLDEQTQQAPHLMPPPFLVDVDGNPHPPRYQRLVPGRQGCSL   618
Phip Lepisosteus oculatus           QMFFHTDYRPLIRDANNYVLDEQTQQAPHLMPPPFLVDVDGNPHPARFQRLVPGRENCRD   614
Phip Danio rerio                    QMFFHTDYRPLIRDAHNFVLDEQTQQAPHLMPPPFLVDVDGNPHPPRFQRLVPGRENCRE   615
Phip Latimeria chalumnae            QMFFHTDYRPLIRDANNFVIDEQTQQAPHLMPPPFLVDVDGNPHPPRYQRLVPGRENCRD   618
Phip Xenopus tropicalis             QMFFHSDYRPLIRDANNYVLDEQTQQAPHLMPPPFLVDVDGNPHPTRYQRLVPGRENCRD   613
Phip Gallus gallus                  QMFFHSDYRPLIRDANNFVLDEQTQQAPHLMPPPFLVDVDGNPHPARYQRLVPGRENCRE   616
Phip Mus musculus                   QMFFHSDYRPLIRDANNFVLDEQTQQAPHLMPPPFLVDVDGNPHPSRYQRLVPGRENCRE   614
Phip Homo sapiens                   QMFFHSDYRPLIRDANNFVLDEQTQQAPHLMPPPFLVDVDGNPHPSRYQRLVPGRENCRE   614
                                    : ***:*****:**  . *:****: **: ********:******   ******: .

Phip Nematostella vectensis         TARQPPTTP-RAELPPLLAEIEA------------AENAALRALIKIFWSLGEL--TGEG   653
Phip Strongylocentrotus purpuratus  NALVPQIGVAENGKLRLLENEAN------------QAASLLDELILRMQQDQDEERGDAP   667
Phip Ciona robusta                  DALVPVVVAADNGVPLIVNEQPDIVADLSPP---------LNEV----------------   672
Phip Branchiostoma belcheri         DQLIPQVGVAANGQPEVMGPIPEAPAVVRPQNEDEEAANILDNMIAEMQRQQDGEEDAGD   681
Phip Petromyzon marinus             HQLIPQMVE----------------------------GSILDNMILRLQREQDERAGATG   650
Phip Danio rerio                    EQLIPQMGLTSSGLNQVVSQQAA------------EGSSPLDSMIQRLQQEQDQRLGSDS   663
Phip Lepisosteus oculatus           EQLIPQMGMTSSGLNQVLSQQVN------------DGSSPLDTMIQRLQQEQDQRLSGDA   662
Phip Xenopus tropicalis             EQLIPQMGLTSSGMNQVLSQQSD------------NEVSPLDSLIQRLQLEQDQRMNADT   661
Phip Latimeria chalumnae            DQLIPQMGVTSSGLNQVLSLQAN------------N--SLLDSMIQRLQQEQDQRLSGEV   664
Phip Gallus gallus                  EQLIPQMGVTSSGLNQVLSQQAN------------QEVSPLDSMIQRLQQEQDQRRSGEA   664
Phip Homo sapiens                   EQLIPQMGVTSSGLNQVLSQQAN------------QEISPLDSMIQRLQQEQDLRRSGEA   662
Phip Mus musculus                   EQLIPQMGVTSSGLNQVLSQQAN------------QDISPLDSMIQRLQQEQDLRRSGEA   662
                                        *                                   *  :

Phip Nematostella vectensis         ALIGEG----ALKLFWREISASEGPGGSRNYGMRQSGDVEGVRQAHGNVPVIEDVSEADI   709
Phip Strongylocentrotus purpuratus  PHEGPR----TPGR----SANGPLSPSMSPRGLRRAGEIEGVRQATGNVPVSQRASQADI   719
Phip Branchiostoma belcheri         AQ---------------AVRNDDTGLRSPPVGLRRSGDVEGVRRAHGNVTVSQQASDSDL   726
Phip Ciona robusta                  ---------RAPGV----QSPTVSPSRNTPLGLRREGEVVGVRNS--SSPRSEIASAADL   717
Phip Petromyzon marinus             QDEPSSELSPVPLTVHPPPSSESEAPRRSPGPRRRSGQVEGVRQNVAQSR--FNTASGDL   708
Phip Lepisosteus oculatus           GSSSLNRG--NRGS----VGSTSEVHSPPNVGLRRSGQIEGVRQMHSNAPRSEIATERDL   716
Phip Danio rerio                    R--------ANRGS----VSSPTEVTSPPNVGLRRSGQIEGVRQMHSNAPRSEIATERDL   711
Phip Latimeria chalumnae            GTSSLNRGNRVLGS----VSSTSEVHSPPNVGLRRSGQIEGVRQMHSNAPRSEIATERDL   720
Phip Xenopus tropicalis             SGGSSSRM--GRAS----VSSSSEVHSPPNIGLRRSGQIEGVRQMHSNAPRSEIATERDL   715
Phip Gallus gallus                  GTSSTSRI--SRAS----VSSTSEVHSPPNIGLRRSGQIEGVRQMHSNAPRSEIATERDL   718
Phip Mus musculus                   GVSNASRV--NRGS----VSSTSEVHSPPNIGLRRSGQIEGVRQMHSNAPRSEIATERDL   716
Phip Homo sapiens                   VISNTSRL--SRGS----ISSTSEVHSPPNVGLRRSGQIEGVRQMHSNAPRSEIATERDL   716
                                                                     *: *:: ***.   .      .:  *:

Phip Nematostella vectensis         TAWRKRKIAPELKPSVAKFEEDRRIHLGRNELMNYLREKKKRPQTAAAKAIQEAV--ALN   767
Phip Strongylocentrotus purpuratus  LSFKTRMVVPALSRAVARFQEEFRRKLDEEASGRFKAERKKKVLPSPVETTDSTDSYERL   779
Phip Branchiostoma belcheri         VAWSKRVVVPDMEPALLESDERRRVRLGEEELSRYKMERKRRPLPAAISRNDSSDS----   782
Phip Ciona robusta                  LVVQRRELVPLLKTAVRMKRDEIARETGFNEISIYNEEITRKNHKSLNKHRAASMNYLNS   777
Phip Petromyzon marinus             LAWNNRLVVPEVAPGLSSTQEGYRHAKGVEESDIYEREKRRKPVSAAHRSDS--PNVA--   764
Phip Lepisosteus oculatus           VAWSRRVLVPELPPAIASKQEKWRTAKGEEEIRIYKSEKKKRALPSQIQREAKH------   770
Phip Danio rerio                    VAWSRRVLVPELSLAIACKQEIWRTAKGEEEINIYRSERRRRSLHSQYHRENRHA-----   766
Phip Latimeria chalumnae            AAWSRRVVVPELPAGVARKQEQWRTAKGEEEVKLYRSEEKKKTAPSHTPREPKS------   774
Phip Xenopus tropicalis             VAWSRRVVVPELSTGVLSKQEEWRTAKGEEEIRMYKAEDKKKTITNHAVRESKV-SYSKN   774
Phip Gallus gallus                  VAWSRRVVVPELSSGVASRQEEWRTAKGEEEVRVYRSEEKRKHVMSHIQRENKIPTFSKN   778
Phip Mus musculus                   VAWSRRVVVPELSAGVA-RQEEWRTAKGEEEIKSYRSEEKRKHLT--VAKENKILTVSKN   773
Phip Homo sapiens                   VAWSRRVVVPELSAGVA-RQEEWRTAKGEEEIKTYRSEEKRKHLT--VPKENKIPTVSKN   773
                                         * :.* :  .:    :      . :    :  *  ::

Phip Nematostella vectensis         ERSQRAQSRRSSRQASRARERARTRARTQRAMYATRSAMSV-------------------   808
```

```
Phip Strongylocentrotus purpuratus   ------RRR------------KKPKKTANHNYRTRANQDGTPLEHGIGMEEEEERRRRQ    820
Phip Branchiostoma belcheri          ---ERKLRNR------------KGHRQTLTHAYRTRTNMAMDTSHSRSR----------S    817
Phip Ciona robusta                   QVN-----REKTR----NS-DYLRKIPSAHHSYSTRLNSMPL------------------    809
Phip Petromyzon marinus              ---AKGLKQRRKQ-----------ASHGGLHGYRTRSTLEEPGRPAPLR-----------    799
Phip Lepisosteus oculatus            HVQEPAGEGGEGR-----------RPQPNQHNYHTRATVEETGRQADTA-----------    808
Phip Danio rerio                     ----IENAAEEGR-----------RHQGNQHNYHTRLAVEETSRQSEPN-----------    800
Phip Xenopus tropicalis              HSLELLLDIEDPK-----------RQSSNSYNYRTRAAPEEQAVPTEDV-----------    812
Phip Latimeria chalumnae             QTSERSVDFGEMK-----------KQQANQHNYRTRSAMEEPVKQTEEA-----------    812
Phip Gallus gallus                   HSHDHLLDTNDSR-----------KQQANQHNYRTRYAVEEPARPAEEL-----------    816
Phip Mus musculus                    HAHEHFLDLGDSK-----------KQQANQHNYRTRSALEETPRPLEEL-----------    811
Phip Homo sapiens                    HAHEHFLDLGESK-----------KQQTNQHNYRTRSALEETPRPSEEI-----------    811
                                                                     * **

Phip Nematostella vectensis          ---EETEE--SGEDEVIVT-----PSDSEDEEEWNES---SRYESS--------------    841
Phip Strongylocentrotus purpuratus   RVGEEGEDTLSDMDEPVDEGAMTGAETSEEEVEWRSTSE-HESESS--------------    865
Phip Branchiostoma belcheri          RQVPEFDS--SSEVELEMEQGGSGTETSEDDQVWDSSSD-SESESS--------------    860
Phip Ciona robusta                   --------------------------SNDEQDWETTTDASSAEEPDAASVENYHANINT    842
Phip Petromyzon marinus              --RAAYDTS-SDENRTG---GPSGASSSDDGDEWQSESSTTSESSS--------------    839
Phip Lepisosteus oculatus            --EEENSSS-EGEGDARPVNGDT--SDE-EGKAWPDDSSSSSDYSS--------------    848
Phip Danio rerio                     --EEEHSAS-EGETEVRPPSRESSDEEREEKEPWPDENSSSSDYSS--------------    843
Phip Xenopus tropicalis              --ENSSSDEAYVESEAVAASGAT--SEEDEKQEWNSDGS-SSDYSS--------------    853
Phip Latimeria chalumnae             --EDETSSS--EEGEVVAANGGT--SEEGE-KVWHSDSSSSSDYSS--------------    851
Phip Gallus gallus                   --ENGHSSS--DEGEVVVASGGT--SEDDE-RAWHSDGS-SSDYSS--------------    854
Phip Mus musculus                    --ENGTSSS--DEGEVLAVSGGT--SEEEE-RAWHSDGS-SSDYSS--------------    849
Phip Homo sapiens                    --ENGSSSS--DEGEVVAVSGGT--SEEEE-RAWHSDGS-SSDYSS--------------    849
                                                                  :   *          .

Phip Nematostella vectensis          --------EY-SDWTEEV--GIRSQPQNDNRR-----TRVRRLLSSSDDETYTDQPGPSS    885
Phip Strongylocentrotus purpuratus   --------EY-SDWTADA--GMNLQPPKKKTR-----RKTLPWESGGESSA---------    900
Phip Branchiostoma belcheri          --------EY-SDWTQDV--GLPKERSRGEKQSQRQRKAPRRPLDSSEEEE---------    900
Phip Ciona robusta                   RKRRMRSDSYSEGWSSEENEGSPVHSNNARKR-----RK------CSEETSICDVT----    887
Phip Petromyzon marinus              --------EY-SDWTAQA--GVNLQPPKRAVR-----RRAAQRGRGSSSDG---------    874
Phip Danio rerio                     --------DY-SDWTADA--GINLEPPKKTSV-----KKKKKKNASSSEEDG---EKKKD    884
Phip Lepisosteus oculatus            --------DY-SDWTADA--GINLQPPKKTVR-----HKKKNTSSSEE---DGDKKKEKE    889
Phip Latimeria chalumnae             --------DY-SDWTADA--GINLQPPKKAPR-----HKTKKPESSSEDEEETEKQKQKP    895
Phip Xenopus tropicalis              --------DY-SDWTADA--GINLQPPKKILK-----HRTKKAESSSEDEENSDKFKQKQ    897
Phip Gallus gallus                   --------DY-SDWTADA--GINLQPPKKIPK-----LKTKKAESSSEDEEGPEKQ-KKQ    897
Phip Mus musculus                    --------DY-SDWTADA--GINLQPPKKVPK-----HKTKKPESSSDEEEESENQKQKH    893
Phip Homo sapiens                    --------DY-SDWTADA--GINLQPPKKVPK-----NKTKKAESSSDEEEESEKQKQKQ    893
                                             .* ..*: :   *   .  .                   .

Phip Nematostella vectensis          PQ----RSQPKKEKKEKPPQKKKPK--------KPKLD------NIDDVLQFYNPPEWLM    927
Phip Strongylocentrotus purpuratus   --------SPSKT-PSATPVKSTRKRRPPK-ALIDDMEIDNDKESMSELPEELKPPLWIT    950
Phip Branchiostoma belcheri          --------EEDEERPKPPPKSKKPKRKNKKPAPVIDMD---QQMSLDDIPDEYLPPKWIT    949
Phip Ciona robusta                   ------TETLDENQP-TTSTNISPKQTVVKPI-----EPPFPIVPGQPPPDEYLPPKWIS    935
Phip Petromyzon marinus              ----ERAGAPDESPPGGERPRRKTRTRRQKRV-AAQLL------GQGRPAEEWLPSVWVT    923
Phip Lepisosteus oculatus            NKKERKNEKRDKDG--ALPKKKKPKERRMKRVPVEDFQ------EQGLTLEEWLPSAWIT    941
Phip Danio rerio                     SKKERRKEKPDKDG--ALPKKKKPKEKR---KRIPELQ------NQGLTLEEWLPSNWIT    933
Phip Xenopus tropicalis              NKKERKKGMEDKDGS-SSPKKKKPKERKQKRLPIGELT------EQGLSLEEWLPSAWIT    950
Phip Latimeria chalumnae             NKKEKKKGSEDKNGMPASPKKKKSKERKRKMLPVGDLP------EQGLTLEEWLPSVWIT    949
Phip Gallus gallus                   IKKERKKGSEEKDGPSTSPKKRKPKERKQKRLAVGVLA------ENGLTLEEWLPSAWIT    951
Phip Mus musculus                    IKKERKKANEEKDGP-TSPKKKKPKERKQKRLAVGELT------ENGLTLEEWLPSAWIT    946
Phip Homo sapiens                    IKKEKKKVNEEKDGP-ISPKKKKPKERKQKRLAVGELT------ENGLTLEEWLPSTWIT    946
                                               .:          . :                         :   *  *:

Phip Nematostella vectensis          CTQPCLSPYVPQIGDEVYYLRQGHERYVKEVMRRKIYDTNPKK-QCYYKLGLEAVELCRI    986
Phip Strongylocentrotus purpuratus   DTTPRRCPYVPQMGDEVMYFRQGHEAYIAAVKKRKVYHIEPKK-QHWTKINLKDHELVRV    1009
Phip Branchiostoma belcheri          DMKPRRSPYVPQVGDEVVYFRQGHELYVDAVRRHKVYEINPRK-QPWHKIELREQEIVRV    1008
Phip Ciona robusta                   QDVARPSPYMPQIGDCVVYFQQGHQLYIKAVLDKNIWNVGPESNICYNMMELNYIEVCNV    995
Phip Petromyzon marinus              HTVPQRCPFVPQMGDEVMYFRQGHEAYVEAAKRNKVQRINPRK-QPWHKIQLREQELVKI    982
Phip Lepisosteus oculatus            DTVPRRCPFIPQMGDEVYYFRQGHEAYVEMAKKNKIFSINPKK-QPWHKMELREQELMKI    1000
Phip Danio rerio                     DTMPRRCPFTPQMGDEVYYFRQGHEAYVEMAKKNKIFSINPKK-QPWHKLELREQELLKI    992
Phip Latimeria chalumnae             DTMPRRCPFVPQMGDEVYYFRQGHEAYVEMAKKNRIYSINPKK-QPWHKMELREQELMKI    1008
Phip Xenopus tropicalis              DTVPRRCPFVPQMGDEVYYFRQGHEAYVEMAKKNKIYRINPKK-QPWHKMELREQELMKI    1009
Phip Gallus gallus                   DTVPRRCPFVPQMGDEVYYFRQGHEAYVVMAKKNKIYSINPKK-QPWHKMELREQELMKI    1010
Phip Mus musculus                    DTLPRRCPFVPQMGDEVYYFRQGHEAYVEMARKNKIYSINPKK-QPWHKMELREQELMKI    1005
Phip Homo sapiens                    DTIPRRCPFVPQMGDEVYYFRQGHEAYVEMARKNKIYSINPKK-QPWHKMELREQELMKI    1005
                                           .*: **:** * *::***: *:  .  ..:    *..   :  : *.  *: .:

Phip Nematostella vectensis          VSLHFSSSNGVPPTLACLKLALISPDTGIHTGASFTVKYHDMQDVLDFIILRQYYDRSID    1046
Phip Strongylocentrotus purpuratus   MGIKYQV---GPPTLCGLKLGFMDPETRKSTGGSFSMKYHDMPDVIDFLVLRQHFDQSVA    1066
Phip Branchiostoma belcheri          IGIRYEI---GPPLLCCLKLGLINPETGVLTGGSFTIRYHDMPDVIDFLVLRQVYQQSVE    1065
Phip Ciona robusta                   TSIEYEI---GPPTLCCLQLQRQNV--KENEEEYFKLRFHDLPDVIDFIVLQNKFDISML    1050
Phip Petromyzon marinus              VGIKYEV---GPPTLCCLKLAFLDADTGKLTGGAFAIKYHDMPDVIDFLVLRQTYDEARA    1039
Phip Lepisosteus oculatus            VGIKYDV---GLPTLCCLKLAFLDPDTGKLTGGSFSMKYHDMPDVIDFLVLRQQFDDARN    1057
Phip Danio rerio                     VGIKYEV---GLPTLCCLKLAFLDPDSGKLTGGSFSIKYHDMPDVIDFLVLRQQFDNARQ    1049
Phip Latimeria chalumnae             VGIKYEV---GLPTLCCLKLAFLDPDTGKLTGGSFTMKYHDMPDVIDFLVLRQQFDEARN    1065
Phip Xenopus tropicalis              VGIKYEV---GLPTLCCLKLAFLDSDTGKLTGGAFTMKYHDMPDVIDFLVLRQQFDEARR    1066
Phip Gallus gallus                   VGIKYEV---GLPTLCCLKLAFLDPDTGKLTGGSFTMKYHDMPDVIDFLVLRQQFDDAKH    1067
Phip Mus musculus                    VGIKYEV---GLPTLCCLKLAFLDPDTGKLTGGSFTMKYHDMPDVIDFLVLRQQFDDAKY    1062
Phip Homo sapiens                    VGIKYEV---GLPTLCCLKLAFLDPDTGKLTGGSFTMKYHDMPDVIDFLVLRQQFDDAKY    1062
                                      .:.:.      * *.  *:*   .          * :::**: **:**::*:: :: :

Phip Nematostella vectensis          RNWQEGDRFRSIIDDSWWAGVITERSPLQEEYPDSQFMCLTVEWDTGETERMNPGNGDET    1106
Phip Strongylocentrotus purpuratus   MKWKLGDRFRAMIDDAWWQGTIVAHSPLDPEHPDSLFQCYLVDWDNGECERMSPWDLEPI    1126
Phip Branchiostoma belcheri          ANWQVGDRFRAIIDDQWWFGTIECQEPFQEEHPDSHFQCFNVCWDTGETERMSPWDLEGV    1125
Phip Ciona robusta                   TKWKPGDRFRSIIDDEWWFGSVVNHQPLDDKYPESPFQSLLVEWDSNEVERMSPWDLEPT    1110
Phip Petromyzon marinus              RNWQVGDRFRAVIDEAWWYGTVLRQEPFQAQYPDSPFQCYAVCWDNEETERMSPWDMEVI    1099
Phip Danio rerio                     RNWMIGDRFRAVIDDAWWFGTIESQEPYQAEYPDSLFQCYNVCWDNGDTEKMSPWDMEEV    1109
Phip Lepisosteus oculatus            RQWSIGDRFRSVIDDAWWFGTIESQEPFQTQYPDSLFQCYNVCWDNGDTEKMSPWDMEII    1117
Phip Xenopus tropicalis              RQWSIGDRFRSVIDDAWWFGTIESQEPLHQDYPDSLFQCYNVCWDNGDTEKMSPWDMELI    1126
Phip Latimeria chalumnae             RQWNIGDRFRSVIDDAWWFGTIESQEPLQLEYPDSLFQCFNVCWDNGDTEKMSPWDMEII    1125
Phip Gallus gallus                   RRWNIGDRFRSVIDDAWWFGTIESQEPLQPDYPDSLFQCYNVCWDNGDTEKMSPWDMELI    1127
Phip Mus musculus                    RRWNIGDRFRSVIDDAWWFGTIESQEPLQPEYPDSLFQCYNVCWDNGDTEKMSPWDMELI    1122
Phip Homo sapiens                    RRWNIGDRFRSVIDDAWWFGTIESQEPLQLEYPDSLFQCYNVCWDNGDTEKMSPWDMELI    1122
                                      .*  *****::**: ** * :  :.* . .:*:* * .  * **. : *:*.* : :

Phip Nematostella vectensis          TSG-----AATSNIPLTDEDRQALA-YEPSEADWFGE------GRDEMTSRIMGCVDRLC    1154
Phip Strongylocentrotus purpuratus   DE-KNMPESKGGSIPVTDDELLAML-YVPGPGEWGET------DRDTCCDRLLKCLQQVM    1178
Phip Branchiostoma belcheri          PAEMDLPDDAHIGIEITADDWKAMW-YNPKESEWSSE------GREPDCQRIIACIEEVM    1178
Phip Ciona robusta                   TEGMVGRSENDRGLPIEEEELKDIFHQRPSASEWRKVDKLYVDSVDQDCSRISTGIEKLL    1170
Phip Petromyzon marinus              PDDARPPPEPGSSVPLTSEERESLL-YRPQDGEWGSE------PRGQQRDRITAALDQLM    1152
```

```
Phip Danio rerio                    PDEVVFPEELGLSMALTQDEQKALL-YKPLDGEWGSR------TRNQECERIIRGINQLC    1162
Phip Lepisosteus oculatus           PNDAVFPEELGLSVPLTEEEHRQLL-YVPLEGEWGSR------TRDEECERIIAGINQLM    1170
Phip Xenopus tropicalis             PDNAVFPEELGTSVPLIEEEVRSLL-YRSLDGEWGCR------SRDEECDRITAAINQLM    1179
Phip Latimeria chalumnae            PSNAVFPEELGTSVPLSDAEHRTLL-YKPLDGEWGSR------TREQEFERIIAEINQLM    1178
Phip Gallus gallus                  PTNAVFPEELGTSVPLTDEERRTLL-YKPLDGEWGSR------TRDQECDRIIAGINQLM    1180
Phip Homo sapiens                   PNNAVFPEELGTSVPLTDGECRSLI-YKPLDGEWGTN------PRDEECERIVAGINQLM    1175
Phip Mus musculus                   PNNAVFPEELGTSVPLTDVECRSLI-YKPLDGEWGAN------PRDEECERIVGGINQLM    1175
                                                .: :   :   :       .:*               .*:   ::.:

Phip Nematostella vectensis         ELSLAGPFVYPVDVQAYPDYWSVVPYPIDLHTIIEKLGNRFYRRANALIWDIKQIERNAR    1214
Phip Strongylocentrotus purpuratus  ELRIAEQFNTPVDLSRYPSYAYMVPYPTDLSTIMRRLERRFYRRITALQWEVRKLEQNAL    1238
Phip Branchiostoma belcheri         SLAPAEAFNAPVDLNCYPMYCMVVEYPTDLTTIKTRLENRWYRRLAALHWEVRRIEQNAE    1238
Phip Ciona robusta                  GLDMFSPFSAPVDLTIYQAYTLAVAYPTDLKLILRRLQNRFYRSLESLLWDVQKLEENAL    1230
Phip Petromyzon marinus             TLDVTAQFATPVDLQAYPLYCTVVAYLTDLSTIRKRLDNGFYRRLSSLMWEVRYIEHNAR    1212
Phip Lepisosteus oculatus           TLDIAAPFVFPVDLHAYPTYCTVVAYLTDLSTIKQRLENRFYRRMSSLMWEVRYIEHNAQ    1230
Phip Danio rerio                    TLDVAKPFELPVDLQAYPTYCTVVAYVTDLSTIRQRLENRFYRRMSSLMWEVRYIEHNAQ    1222
Phip Latimeria chalumnae            TLDIASPFVAPVDLQAYPMYCTVVAYPTDLSTIKERLENRFYRRLSSLMWEVRYIEHNAR    1238
Phip Xenopus tropicalis             TLDIASAFVAPVDLQAYPMYSTVVAYPTDLSTIKQRLENRFYRRLSSLMWEVRYIEHNTR    1239
Phip Gallus gallus                  TLDIASAFVAPVDLQAYPMYCTVVAYPTDLSTIKQRLESRFYRRLSSLMWEVRYIEHNTR    1240
Phip Mus musculus                   TLDIASAFVAPVDLQAYPMYCTVVAYPTDLSTIKQRLENRFYRRFSSLMWEVRYIEHNTR    1235
Phip Homo sapiens                   TLDIASAFVAPVDLQAYPMYCTVVAYPTDLSTIKQRLENRFYRRVSSLMWEVRYIEHNTR    1235
                                     *     *  ***:  *  *   * *  **  *  :*   :**   :* *::: :*.*:

Phip Nematostella vectensis         LYNEEDSQIVNNSTRLVNILSEFIRDSTCRDVLSLVGEE------------EE----LA    1257
Phip Strongylocentrotus purpuratus  EFNERDSIIVQWAETMVKILMEVTKDDHCDDAMDIYNRITKDSDVDVCVDDSHS----GS    1294
Phip Branchiostoma belcheri         KFNESDSQIVKSARLITKILQEVIKDNSCTDAIAVFNRFAGEQEEVDVEGDAEE----ED    1294
Phip Ciona robusta                  AFNEPDSDIVKKATILHSLLGEFINNQSCHNIEDIYATHGVDHIVDIVEPACESDHSVEC    1290
Phip Petromyzon marinus             AFNEPGSPIVKSAKHVSDLLLHFIKDSSCTDILTLHNARKKDEPEEE---SEEE----HE    1265
Phip Lepisosteus oculatus           TFNEPGTPIVKTAKFVTDLLLQFIKDQSCTDIIPLYNSMKKKTFSDTEEDE--------D    1282
Phip Danio rerio                    TFNEPGAFIVRTAKFVSDLMLQFIKDQSCTDIIPLYNSMKKTAFSDSSDDEDED----ED    1278
Phip Latimeria chalumnae            TFNEPGSPIVRTAKFVTDLLLQFIKDQTCSDIIPLYNSMKKKMLSDSEDEDE-------E    1291
Phip Xenopus tropicalis             TFNEPGSPIERSAKLVIDVLLHFVKDQCCYDIIPLYNNLKKKLLSDSEEEE--------E    1291
Phip Gallus gallus                  TFNEPGSPIVKSAKFVTDLLLHFIKDQSCYDIILLYNAMKKKVLSDSDEEE--------E    1292
Phip Mus musculus                   TFNEPGSPIVKSAKFVTDLLLHFIKDQTCYNIIPLYNSMKKKVLSDSEE-E--------E    1286
Phip Homo sapiens                   TFNEPGSPIVKSAKFVTDLLLHFIKDQTCYNIIPLYNSMKKKVLSDSED-E--------E    1286
                                     :** .: * . :  : .:: .. .:. * :   :

Phip Nematostella vectensis         EEIEVPYKHRR---------QNKRQKIQE--EEEPREEVHVNHSWVTSARILLNFLMSRE    1306
Phip Strongylocentrotus purpuratus  E--DKRGSSSR---------SKGKKGSPSKKQKRKNRACYNSKAWIEQCKKLLKDMWDCH    1343
Phip Branchiostoma belcheri         EE---EEQHEN---------KKKKQSSSKSRPRPPSQDPPDPHMWKRQCRELLNIMFQME    1342
Phip Ciona robusta                  DVVTEPTTSDRLNSITNVITRAKKSRYQPWISEDEKEDQDRMDEWKGSCSNLLSFAWDTE    1350
Phip Petromyzon marinus             EDREAPSTSTG---------RLRKRRT----QRYRRESAYNIAAWKQQCRQLVSVILERE    1312
Phip Lepisosteus oculatus           EDTDAPGTSTR---------KKK-EHKPMIRKLRNKAQSYDIKAWKTRCKELLDLIFQCE    1332
Phip Danio rerio                    EEHEAPCTSNR---------NKKRQQQQPMLRSLRSKPSSDPQAWKERCRELLELIFQCE    1329
Phip Latimeria chalumnae            NDAVAPGTSTR---------KRK-QEHQPRRKLRNRAQSYDIQAWRRQCQELLDLIFQCE    1341
Phip Xenopus tropicalis             KDADVPGTSTR---------KR--KEYQPRRKLRSRAPSYDLHSWKKQCEELLNLIFQCE    1340
Phip Gallus gallus                  KDTNVPGTSTR---------KR--KDHQPKRRLRNRAQSYDIQSWKKQCQELLNLIFQCE    1341
Phip Mus musculus                   KDADVPGTSTR---------KR--KDHQPRRRLRNRAQSYDIQAWKKQCQELLNLIFQCE    1335
Phip Homo sapiens                   KDADVPGTSTR---------KR--KDHQPRRRLRNRAQSYDIQAWKKQCEELLNLIFQCE    1335
                                    .                                           *   .  *:.   . .

Phip Nematostella vectensis         DAEPFLAPVNLADFPDYTDYVEQPMDFSTVWRKFVENQYSDPEQLVSDIRLVFSNSRAYN    1366
Phip Strongylocentrotus purpuratus  DSEPFRTPVDERAYPEYREIIDHPMDLSTLKQQFALGSYRDPMDLGKDVRLMFTNAKSFT    1403
Phip Branchiostoma belcheri         DSEPFRNPVDPLEYPDYRNIIDTPMDLGTVREQLMVGAYENPTELSKDVRLIFNNSKTYT    1402
Phip Ciona robusta                  DASPFREGVNEEHYPDYYDIVEEPMNLTMIREKLSKDGYNNPKEVESDLHLMFTNSKAYN    1410
Phip Petromyzon marinus             DSEPFRQPVDPIAYPDYRTIIPTPMDLGTVRERLREDTYHDPVDFCKDVRLIFSNAKAYT    1372
Phip Danio rerio                    DSEPFRQPVDLDEYPDYLDIVDTPMDFGTVLNRLLAGEYDTPMDLCKDVRLIFSNSKAYT    1389
Phip Lepisosteus oculatus           DSEPFRQPVDLQEYPDYRDIIDTPMDFGTVLNTLQAGDYESPTELCKDVRLIFSNSKAYT    1392
Phip Latimeria chalumnae            DSEPFRQPVDLLEYPDYRDIIDTPMDFGTVKETLEAGNYESPMELCKDVRLVFSNSKSYT    1401
Phip Xenopus tropicalis             DSEPFRQPVDLLEYSDYREIIDTPMDFGTVRETLEAGNYETPLELSKDVRLIFSNSKAYT    1400
Phip Gallus gallus                  DSEPFRQPVDLLEYPDYRDIIDTPMDFATVRETLEAGNYESPMELCKDVRLIFSNSKAYT    1401
Phip Mus musculus                   DSEPFRQPVDLLEYPDYRDIIDTPMDFATVRETLEAGNYESPMELCKDVRLIFSNSKAYT    1395
Phip Homo sapiens                   DSEPFRQPVDLLEYPDYRDIIDTPMDFATVRETLEAGNYESPMELCKDVRLIFSNSKAYT    1395
                                    *:.**   *:   : :*   :  **::  : . :  . *  * :. .*::*:*.*::::.

Phip Nematostella vectensis         TQPRSRIYSMTIRLAAMFEDRVDSIFSDW-------------------------------    1395
Phip Strongylocentrotus purpuratus  PNKKSRIYGMTLRLSAVFEEHFLKIIGSYRSALKYEKQVKTGFKKRKQL--M--------    1453
Phip Branchiostoma belcheri         PNKKSRIYSMTLRLSAFFEENIRQIVAEWRSAKKHVERLAQNRYYRKSQ--E-SGRKAAR    1459
Phip Ciona robusta                  TNKKSKIYSMTVRLQALIRARMQPIIAEYENVVSMRGALTAQLMEKQMSNSYITRSRRRR    1470
Phip Petromyzon marinus             PNKKSRIYGMTLRLSALFEEHIRGIVSEYKSALRYHERRERAAC-----------RGKRA    1421
Phip Lepisosteus oculatus           PSKKSRIYSMSLRLSALFEEHVSAILSDYKSAHRFHSANKL------------SSQRKRR    1440
Phip Danio rerio                    PSKKSRIYSMSLRLSALFEEHISSILTDFKAAQSLHSERLTRQRLHTDR--LTRQSVKKR    1447
Phip Latimeria chalumnae            PSKKSRIYSMNLRLSALFEEHISSILSDYKSALRFHEKSKL------------TRQKKKR    1449
Phip Xenopus tropicalis             PSKRSKIYSMSLRLSAFFEERMSNILADYRSALRFHNRNTK------------TKSRRKR    1448
Phip Gallus gallus                  PSKRSRIYSMSLRLSAFFEEHISSILSDYKSALRFHKRNTI------------KRRRKKR    1449
Phip Mus musculus                   PSKRSRIYSMSLRLSAFFEEHISSVLSDYKSALRFHKRNTI------------SKKRKKR    1443
Phip Homo sapiens                   PSKRSRIYSMSLRLSAFFEEHISSVLSDYKSALRFHKRNTI------------TKRRKKR    1443
                                     . :*:**.*.:** *.:. ..  :. .:

Phip Nematostella vectensis         ------------------------------------------------------------    1395
Phip Strongylocentrotus purpuratus  ----SSPEERHKSPRRHHVRRLNVKRSLTAGHSMDGDDDASDIDIDAPGPSCNL--RQMA    1507
Phip Branchiostoma belcheri         MRRESGASSTSSRRDRK--TGS-KTED--------------------GPSTSS--QRME    1493
Phip Ciona robusta                  KRSSR----YSQPPEDK-----------------------PIDPNEPSSSRAPESRRSN    1502
Phip Petromyzon marinus             KHARSGSSSRDSSPEVR--KRV-KAQTR------------NPNDASSSSSSSSS--SSS-    1463
Phip Lepisosteus oculatus           RRSTSPSSSAASSPERK--RRS-VKAPL-------------KPEPCAVSPSPGP--SRPA    1482
Phip Danio rerio                    RRRSSSHSSSASSPERK--RRV-SSRAP------------PRLDSAAPPTPAGP--SRAP    1490
Phip Latimeria chalumnae            NRSSSVSSSAASSPERK--RRL-LKTEV-------------KADISS-AQSSTP--GRSV    1490
Phip Xenopus tropicalis             NRSSSSSSSPASSPERK--RRY-VKQQP-------------NSENTTMSPASVH--QRTV    1490
Phip Gallus gallus                  SRSSSVSSSVASSPERK--RRL-IKPQL-------------KGEPSASSSSSVS--VRST    1491
Phip Mus musculus                   NRSSSLSSSAASSPERK--KRI-LKPQL-------------KSEVSTSPFS-IP--TRSV    1484
Phip Homo sapiens                   NRSSSVSSSAASSPERK--KRI-LKPQL-------------KSESSTSAFS-TP--TRSI    1484

Phip Nematostella vectensis         ------------------------------------------------------------    1395
Phip Strongylocentrotus purpuratus  RST---KKRRK--EESDEEE-EKEDDEE----------EEEEDDKDKPGTSGCRGNSSRV    1551
Phip Branchiostoma belcheri         ------T---DDEDENDEEE-EK---------------------EEKKKKAGP-SSAQA    1520
Phip Ciona robusta                  SSRRCRILSPDDPSSSDSEDQNPPPRLRK----------------DRPQLAQS--SRLRS    1544
Phip Petromyzon marinus             ------------SDVAGMGV-QASARPSSSSSGAAMMSHGGAAGKGAATTASGR-PSEGP    1509
Phip Lepisosteus oculatus           ------TLRQTVPHVNGRAV-DSPAVVRT-RSTT---RFGNHSGVEPPSTSSGA-KSASP    1530
Phip Danio rerio                    ------SLRQTHPQINGKAE-TPVVPGRT-RSSA---RFGSPITDVAPAQSAPP-PLVES    1538
Phip Latimeria chalumnae            ------VQRHSTAQVNGKAA-ESTLVVRT-RGAR---RVTDAAVTEQPSTSSGA-KSIIS    1538
Phip Xenopus tropicalis             ------AHKVTNSLVNGKAT-EPSTLART-RSYK---RQVEPAVPEKPTTSGTP-KQLTA    1538
```

Phip *Gallus gallus*	------ALRHAPPQMNGKAA-ETSSLVRT-RSNR---GITDVASTEQPSTSSGG-KPLTI	1539
**Phip *Mus musculus***	**------LPRHNAAQMNGKP--ESSSVVRT-RSNR---VAVDPVVTEQPSTSSAT-K----**	**1527**
**Phip *Homo sapiens***	**------PPRHNAAQINGKT--ESSSVVRT-RSNR---VVVDPVVTEQPSTSSAA-K----**	**1527**

Phip *Nematostella vectensis*	--	1395
Phip *Strongylocentrotus purpuratus*	SHPKQLNGYDKDEDDSDTDVEPLANVKLKVDNNNSSKTES---KSGTKGGKAKAKRSSAK	1608
**Phip *Ciona robusta***	**RLPQKQNKFQRSDTT------------------SSSKDSS--------------FLSDPR**	**1572**
Phip *Branchiostoma belcheri*	SQPRRTGKVMKGEEESEEEEEEEKKPVNS-RPSRSS------------------------	1555
Phip *Petromyzon marinus*	GRP------------SRSSAEAGGAAMLS-SSSSSSSTSSSAVGMTTRGGKGRGVAATAA	1556
**Phip *Danio rerio***	**------------------------------SSSRVLRTQN--------------PHSTAV**	**1554**
Phip *Lepisosteus oculatus*	SLP------------------ANRKPVTE-SPSRHLKVHN--------------SASTPS	1557
Phip *Latimeria chalumnae*	KPTA---------------TLPAKS-VTE-NTSKHSKLQN--------------NLSSLN	1567
**Phip *Xenopus tropicalis***	**KS--------------NHSTVAGKSAMIE-NAAKLLKSQN--------------TSP---**	**1566**
Phip *Gallus gallus*	KPLIT------K---PNTTAGSGKS-VLE-NSTKHSKNQN--------------ILPVSS	1574
**Phip *Mus musculus***	**-AFVS------K---TNTSAMPGKA-MLE-NSVRHSKALS--------------TLSSPD**	**1561**
**Phip *Homo sapiens***	**-TFIT------K---ANASAIPGKT-ILE-NSVKHSKALN--------------TLSSPG**	**1561**

Phip *Nematostella vectensis*	--	1395
Phip *Strongylocentrotus purpuratus*	P---------VVKEEEEEEKEKEKEEDSGEETEIDSGEETEIDEQHVKKEKEGDGSGEEE	1659
Phip *Branchiostoma belcheri*	QRNATSGTSK----------VRIKSP--------SSERSDE-------------------	1578
**Phip *Ciona robusta***	**P----------------RRSKPHYLPQSSERMHL----------RNIRK-RRNTTVRYVE**	**1605**
Phip *Petromyzon marinus*	VTNGRAGAAAVGRRGRPAHRVFRCAP--------PGGAEDEGSRPPVCNSKRKSA--EVS	1606
Phip *Lepisosteus oculatus*	F-------------SSDTDNGRSSSV--------A---ETSG-KKKVKR-KQKSS---VE	1588
**Phip *Danio rerio***	**----------------SRENESSAVP--------P---SNGGGARESRR-KLRTP---VK**	**1583**
Phip *Latimeria chalumnae*	H-------------PCPTRNTKISST--------G---ENSEQVKHFKR-RRKSP---IS	1599
**Phip *Xenopus tropicalis***	**N-------------QASNHLIKNNVP--------K---EKLEKDKPLKR-RMKAS---SN**	**1598**
Phip *Gallus gallus*	Q-------------SENSHNTRNNST--------K---ENPEKEKPIKR-KVKSS-----	1604
**Phip *Mus musculus***	**P-------------LTFSHATKNNSA--------K---ENMEKEKPVKR-KMKSS---VF**	**1593**
**Phip *Homo sapiens***	**Q-------------SSFSHGTRNNSA--------K---ENMEKEKPVKR-KMKSS---VL**	**1593**

Phip *Nematostella vectensis*	--	1395
Phip *Strongylocentrotus purpuratus*	EEEEEEEKEEEEEEADDSEEDDSEEEEEEEEEEEEQEEEDEEPGRKTRLRS---------	1710
Phip *Branchiostoma belcheri*	-----------------------------EESEKEQSEEETRPSRTTRSTRSSARELRDS	1609
**Phip *Ciona robusta***	**SL---------SEASDD--------AF-------ERLNDVSSRGRVRRLSSKAMAA-KLS**	**1640**
Phip *Petromyzon marinus*	SS-----SSSSSSSSSSSSSSSSSSKATHGKAENATGKRRHRPGRRS------GAGKQDL	1655
Phip *Lepisosteus oculatus*	HQ--------------------------PVNS-------------------------ELS	1597
**Phip *Danio rerio***	**--------------------------LTPGPV-------------------------DHS**	**1592**
Phip *Latimeria chalumnae*	SP-----QATF---------SKVTSYIYTANS-------------------------KND	1620
**Phip *Xenopus tropicalis***	**-------------------------MALRADR-------------------------KEI**	**1608**
Phip *Gallus gallus*	-------------------------AVNQQDC-------------------------KNN	1614
**Phip *Mus musculus***	**SK-----ASPL---------PKSAAVIEQGEC-------------------------KNN**	**1614**
**Phip *Homo sapiens***	**PK-----ASTL---------SKSSAVIEQGDC-------------------------KNN**	**1614**

Phip *Nematostella vectensis*	--	1395
Phip *Strongylocentrotus purpuratus*	----RRSLTNGNNSR-------RGGRRKARRDEEDSESEEE----------------EKE	1743
Phip *Branchiostoma belcheri*	SSETRTPVANGHTKEYK----TRAKARSQQL--------------------------SSD	1639
**Phip *Ciona robusta***	**--**	**1640**
Phip *Petromyzon marinus*	ADGGGERRLNGHGSRR------------PRASSAV----------------------AST	1681
**Phip *Danio rerio***	**SPPQNSLHLNGHSQVTVG-MVRRGRGRP-RLDTQIE---TPV----DTPPPPLPKTSSKK**	**1643**
Phip *Lepisosteus oculatus*	SEPQNSMQLNGHGGHIPNYVAKRG--RKPKSEAMAR-VENTAKEEPFVKPESLAKPPGKK	1654
**Phip *Xenopus tropicalis***	**LHQASSIQVNGHSSEPLKPIVKGRPGRKPKVILNNS---NN--YETITV----VQVVPKK**	**1659**
Phip *Latimeria chalumnae*	TIASGSLQVNGHNGHPSNSFVKRGPGRRPKIEATIS------------S----CQVLPKK	1664
Phip *Gallus gallus*	ALASGSIQVNGHGGQPSKPPVKRGPGRKPKIEANNS------------T----CEVVHKK	1658
**Phip *Mus musculus***	**VLIPGTIQVNGHGGQPSK-LVKRGPGRKPKVEVNTS------------S----GEVTHKK**	**1657**
**Phip *Homo sapiens***	**ALVPGTIQVNGHGGQPSK-LVKRGPGRKPKVEVNTN------------S----GEIIHKK**	**1657**

Phip *Nematostella vectensis*	--	1395
Phip *Strongylocentrotus purpuratus*	EEEKEEEEEEEEGGDGGESDH-SNYEAGTD--INSNEGSESSSTEESDSDSSDSEVEGHY	1800
Phip *Branchiostoma belcheri*	KGSDDS---SSS-GDSSSS---SSGSSSSDSSSSDSEDSSSSSSDSSDS-ESDG------	1685
**Phip *Ciona robusta***	**--**	**1640**
Phip *Petromyzon marinus*	RAAPAKRLPSSE-------------SESSDDGDDDDDGVGDAAAKK--A-NDDGKNDGDE	1725
Phip *Lepisosteus oculatus*	RGRKSKKELEAM-RESELWDTAEEAEEERDQCASDEAGTSSSVAAVTDT-AADSDLAQPI	1712
**Phip *Danio rerio***	**KERKSKKDTESP-RRNFS------HEEELMLPTGDDGGASSTAQD-----SNLS------**	**1685**
Phip *Latimeria chalumnae*	RGRKPKKVQSSE-LVTEEQHA-AR----V-NKDNDEEAASSTCNSNSDN-NSRKGEVLQK	1716
**Phip *Xenopus tropicalis***	**RGRKPKYPRNEA-LQSVSQKPPNSV---VETGKIKEQPASSSCNSHAAP-CEKTENEV-P**	**1713**
Phip *Gallus gallus*	RGRKPKKLQIVE-QQKVAEQNTVQ----TTKDVLEEASASTACNSLSE--NNMKEDLVQK	1711
**Phip *Mus musculus***	**RGRKPKNLQCAK-QENSEQNNMHP----IR----ADVLPSSTCNFLSET-NAVKEDLLQK**	**1707**
**Phip *Homo sapiens***	**RGRKPKKLQYAK-PEDLEQNNVHP----IR----DEVLPSSTCNFLSET-NNVKEDLLQK**	**1707**

Phip *Nematostella vectensis*	--	1395
Phip *Strongylocentrotus purpuratus*	RTRRRTTNRRTQKSLKKSR----RGQAIGKKRKAKSKHSPRSSPKKRSKVD------YND	1850
Phip *Branchiostoma belcheri*	--KTSR-KRKTQNSSSKKR---------KATSHSNVNGKSRSSKRRK-------------	1720
**Phip *Ciona robusta***	**--**	**1640**
Phip *Petromyzon marinus*	KGRGGG-PPRWPPPRGRL--	1742
Phip *Lepisosteus oculatus*	KSRRGR-KPKGQKKMEDL---PPEPS------TVSPKMQTRSSRRQSESLHEP---PPQL	1759
**Phip *Danio rerio***	**--DSGE-YPKARGRPRITK-T------IDTPAPPSPKTLRRSSRRGNEEITPHTSGSAQS**	**1735**
Phip *Latimeria chalumnae*	KVRRGR-KPKQKNQT--VE--------PKLKEAPSTKMQTRSSRKHTE--------EPME	1757
**Phip *Xenopus tropicalis***	**KKRRGR-KPKVKTEPTSLVAVIPAPSAHELKREETVKIQTRSKCKKSD--------QSEG**	**1764**
Phip *Gallus gallus*	KGRGGR-KPKRKIKTHPSG---------SDLLIPANVKMQTRSRRKKID--------EPME	1754
**Phip *Mus musculus***	**KSRGGR-KPKRKMKTHNLD---------SELIVPTNVKVLRRSNRKKTD--------DPID**	**1750**
**Phip *Homo sapiens***	**KNRGGR-KPKRKMKTQKLD---------ADLLVPASVKVLRRSNRKKID--------DPID**	**1750**

Phip *Nematostella vectensis*	--	1395
Phip *Strongylocentrotus purpuratus*	EDDNYVPSHKVSRQTERRTRNQGRHMQYYGDFDESDNQSGEEENLQSGEEDDGKSVSSRG	1910
Phip *Branchiostoma belcheri*	-VAYEHDSDYDDGDRPYNTRTSNRGTRFYHDHSDVDSD----------TDAVSRSVSRSG	1769
**Phip *Ciona robusta***	**--**	**1640**
Phip *Petromyzon marinus*	--	1742
Phip *Lepisosteus oculatus*	DGEFEEEEEDRGSKSPMKTRNQGRRTAFYNEDDSEEEQR----QLLFEDASLTFGTSSKG	1815
**Phip *Danio rerio***	**EEPEVYIGEDGSSKGQMKTRNQGRRTAFYNEEDSEEEQR----QLLFEDASITFGTSSKG**	**1791**
Phip *Latimeria chalumnae*	EDIRTDETETKGSESHMKTRNQGRRTAFYNEDDSEEEQR----QLLFEDTSLTFGTSSRG	1813
**Phip *Xenopus tropicalis***	**-ESLTVSEDINNTEIQMRTRNQGRRTAFYNEEDSEEEQR----QLLFEDTSLTFGTSSRG**	**1819**
Phip *Gallus gallus*	EG----SEELKDSEPHMRTRNQGRRTAFYNEDDSEEEQR----QLLFEDTSLTFGTSSRG	1806
**Phip *Mus musculus***	**EEEE--FEELKGSEPHMRTRNQGRRTAFYNEDDSEEEQR----QLLFEDTSLTFGTSSRG**	**1804**
**Phip *Homo sapiens***	**EEEE--FEELKGSEPHMRTRNQGRRTAFYNEDDSEEEQR----QLLFEDTSLTFGTSSRG**	**1804**

Phip *Nematostella vectensis*	----------------	1395
Phip *Strongylocentrotus purpuratus*	RVRKLTARARARLLST	1926
Phip *Branchiostoma belcheri*	RIRKLTPRARANLVGF	1785
**Phip *Ciona robusta***	**----------------**	**1640**
Phip *Petromyzon marinus*	----------------	1742
Phip *Lepisosteus oculatus*	RVRKLTEKAKANLIGW	1831
**Phip *Danio rerio***	**RVRKLTEKAKANLIGW**	**1807**
**Phip *Xenopus tropicalis***	**RVRKLTEKAKANLIGW**	**1835**
Phip *Latimeria chalumnae*	RVRKLTEKAKANLIGW	1829
Phip *Gallus gallus*	RVRKLTEKAKANLIGW	1822
**Phip *Mus musculus***	**RVRKLTEKAKANLIGW**	**1820**
**Phip *Homo sapiens***	**RVRKLTEKAKANLIGW**	**1820**
