# Supplementary material for: Gene expression in notochord and nuclei pulposi: a study of gene families across the chordate phylum
Source: BMC Ecol Evol. 2023 Oct 27;23:63. doi: 10.1186/s12862-023-02167-1 (PMC10605842; doi:10.1186/s12862-023-02167-1)
Supplement: Supplementary file 2 — Additional file 2: Figure S2. Comparative view of the genomic context of the single-copy RgmA/B/C/D genes found in hemichordates and tunicates and of RgmB genes of vertebrates. [file 12862_2023_2167_MOESM2_ESM.pdf]

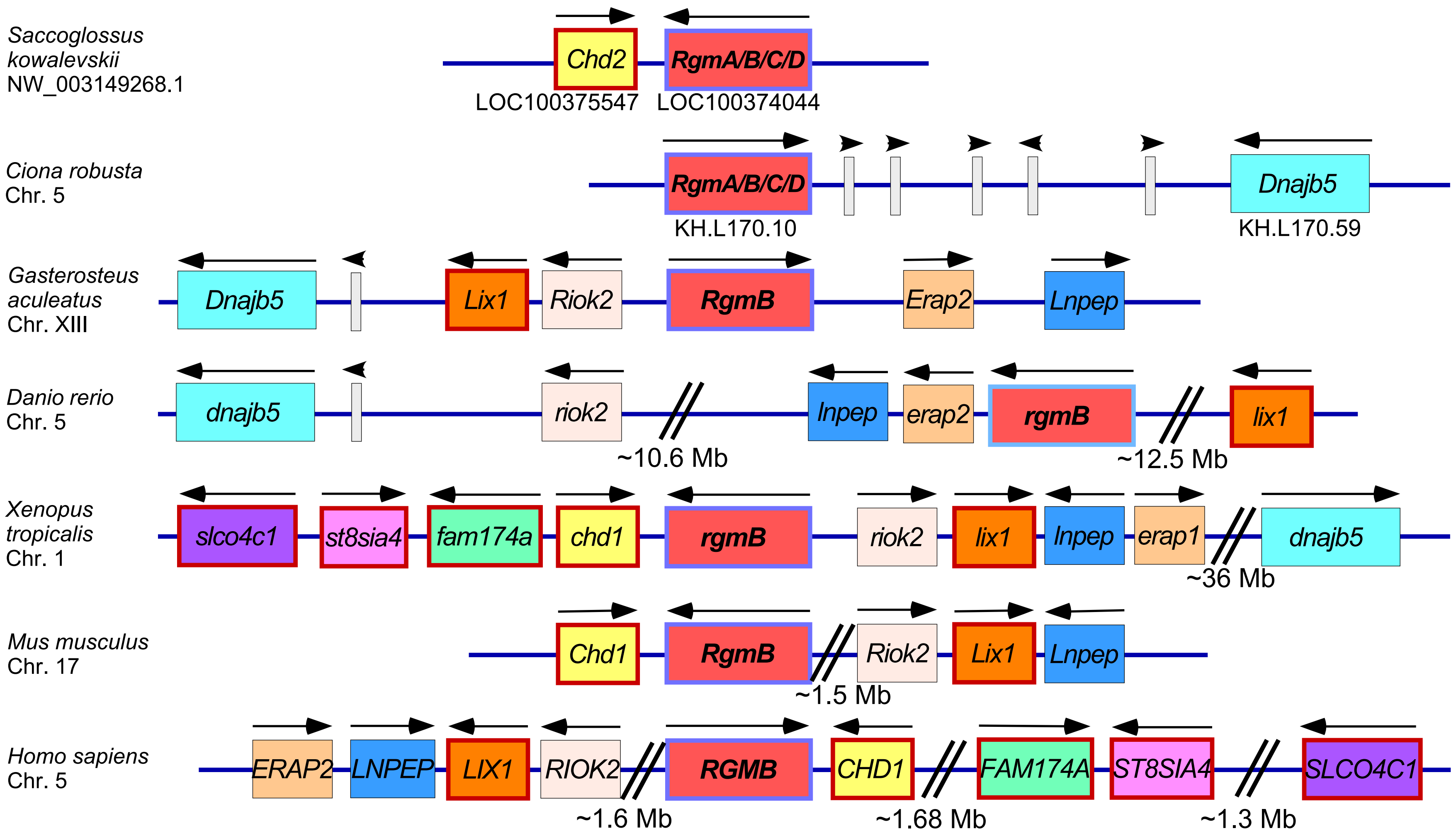
Saccoglossus kowalevskii NW_003149268.1
Chd2
RgmA/B/C/D
LOC100375547
LOC100374044
Ciona robusta Chr. 5
RgmA/B/C/D
Dnajb5
KH.L170.10
KH.L170.59
Gasterosteus aculeatus Chr. XIII
Dnajb5
Lix1
Riok2
RgmB
Erap2
Lnpep
Danio rerio Chr. 5
dnajb5
riok2
~10.6 Mb
lnpep
erap2
rgmB
~12.5 Mb
lix1
Xenopus tropicalis Chr. 1
slco4c1
st8sia4
fam174a
chd1
rgmB
riok2
lix1
lnpep
erap1
~36 Mb
dnajb5
Mus musculus Chr. 17
Chd1
RgmB
~1.5 Mb
Riok2
Lix1
Lnpep
Homo sapiens Chr. 5
ERAP2
LNPEP
LIX1
RIOK2
~1.6 Mb
RGMB
CHD1
~1.68 Mb
FAM174A
ST8SIA4
~1.3 Mb
SLCO4C1
