# Supplementary figures and images for: Gene expression in notochord and nuclei pulposi: a study of gene families across the chordate phylum
Source: BMC Ecol Evol. 2023 Oct 27;23:63. doi: 10.1186/s12862-023-02167-1 (PMC10605842; doi:10.1186/s12862-023-02167-1)

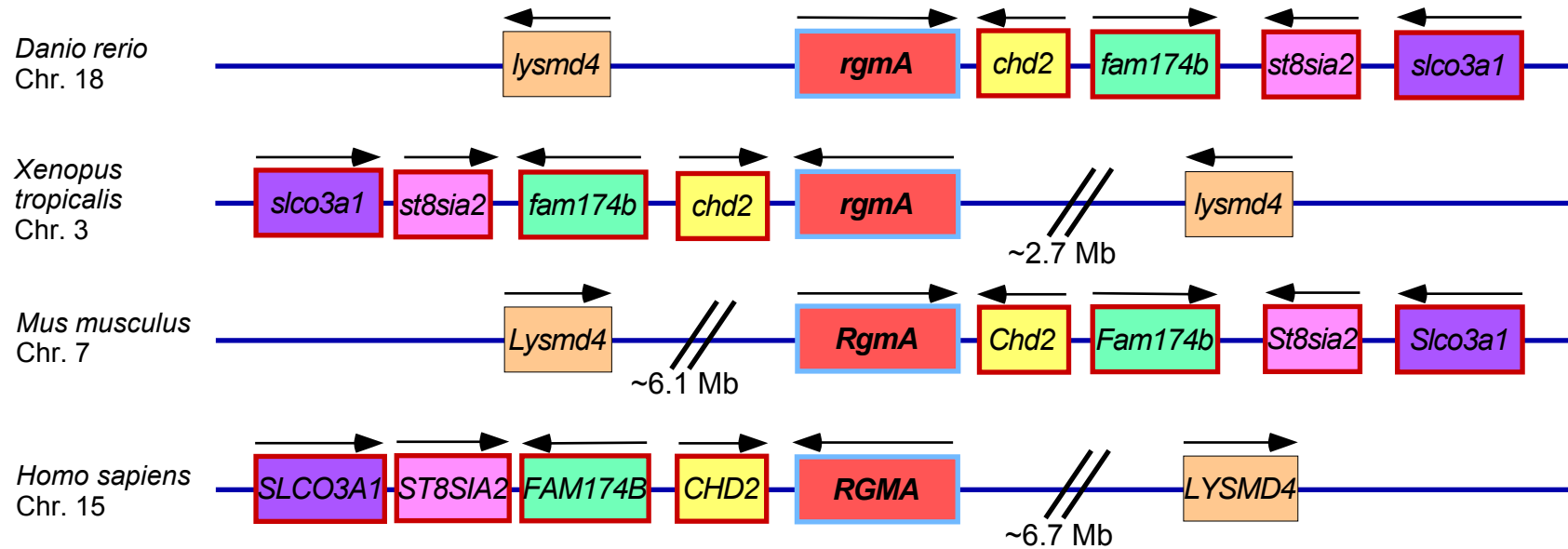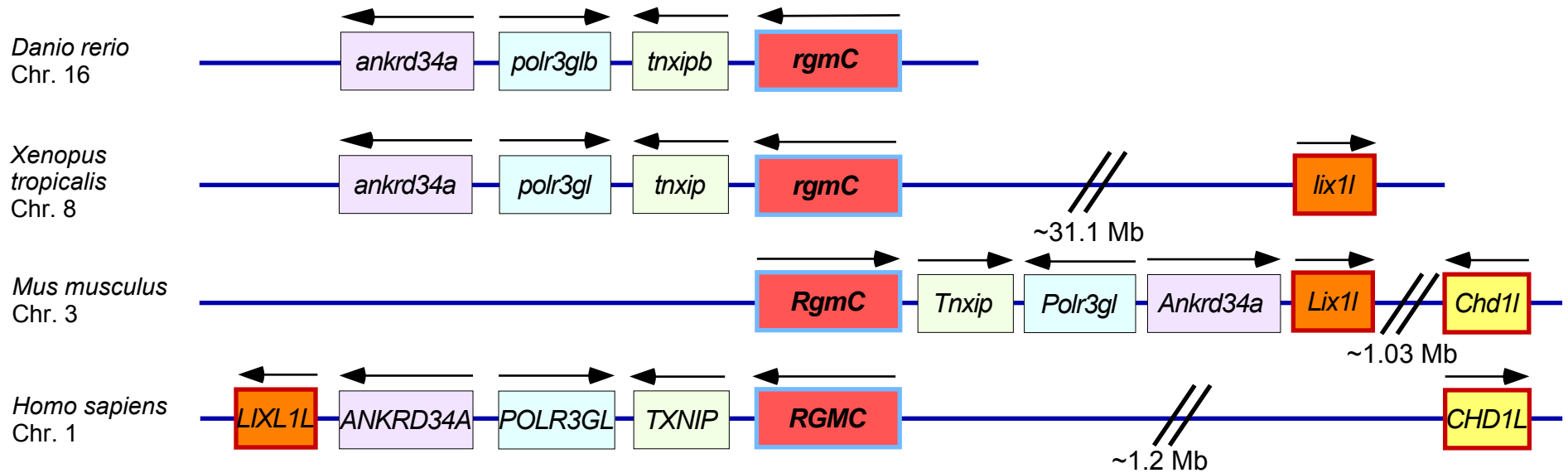

Supplement: Supplementary file 3 — Additional file 3: Figure S3. Comparative view of the genomic context of vertebrate RgmA and RgmC genes. [file 12862_2023_2167_MOESM3_ESM.pdf]

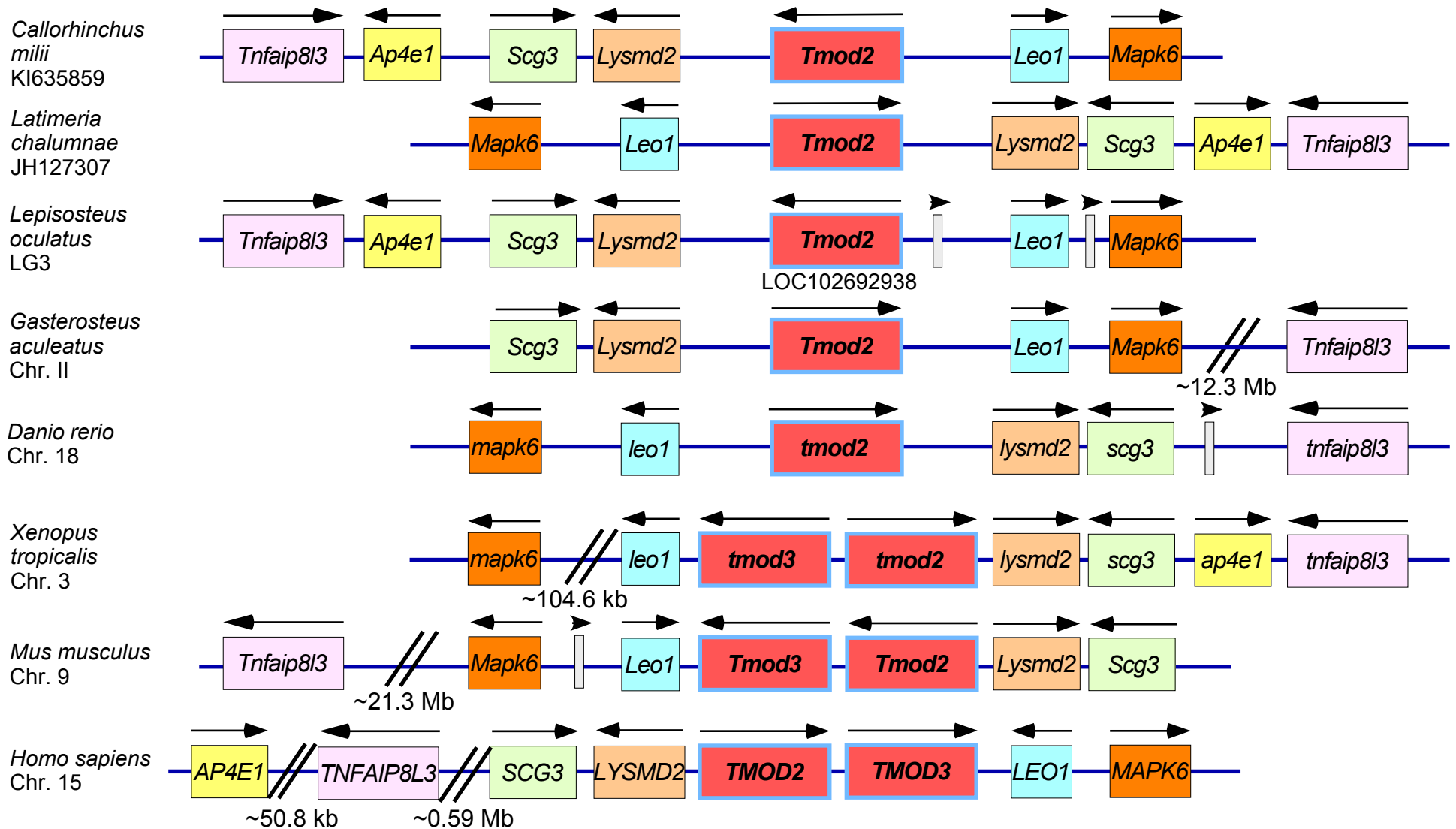

Supplement: Supplementary file 5 — Additional file 5: Figure S5. Comparative view of the genomic context of vertebrate Tmod2 and Tmod3 genes. [file 12862_2023_2167_MOESM5_ESM.pdf]

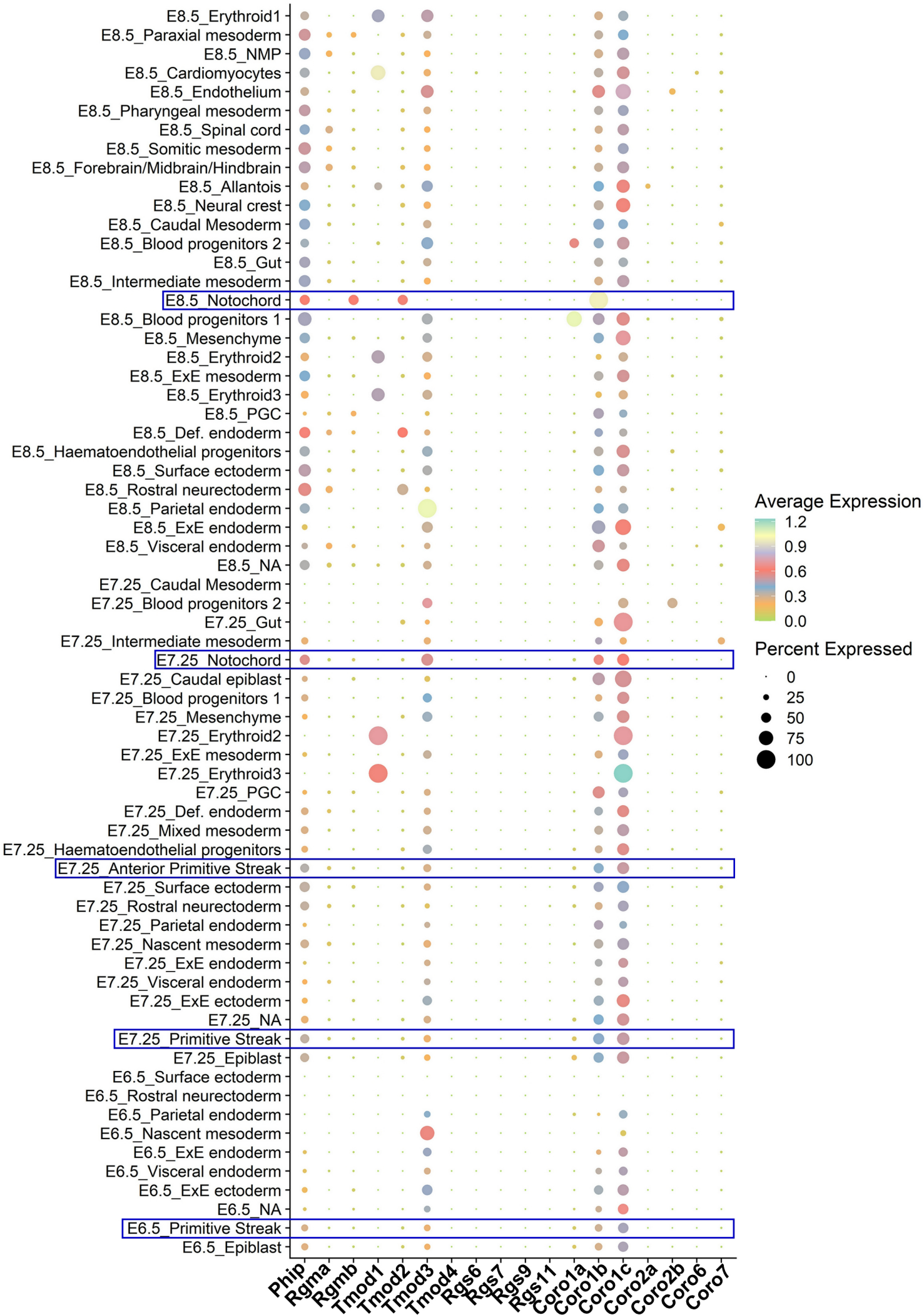

Supplement: Supplementary file 7 — Additional file 7: Figure S7. Expression of members of the gene families analyzed in this study during select stages of mouse gastrulation. [file 12862_2023_2167_MOESM7_ESM.pdf]
