# Supplementary material for: Gene expression in notochord and nuclei pulposi: a study of gene families across the chordate phylum
Source: BMC Ecol Evol. 2023 Oct 27;23:63. doi: 10.1186/s12862-023-02167-1 (PMC10605842; doi:10.1186/s12862-023-02167-1)
Supplement: Supplementary file 4 — Additional file 4: Figure S4. Comparative view of the genomic context of the fish-specific RgmD genes. [file 12862_2023_2167_MOESM4_ESM.pdf]

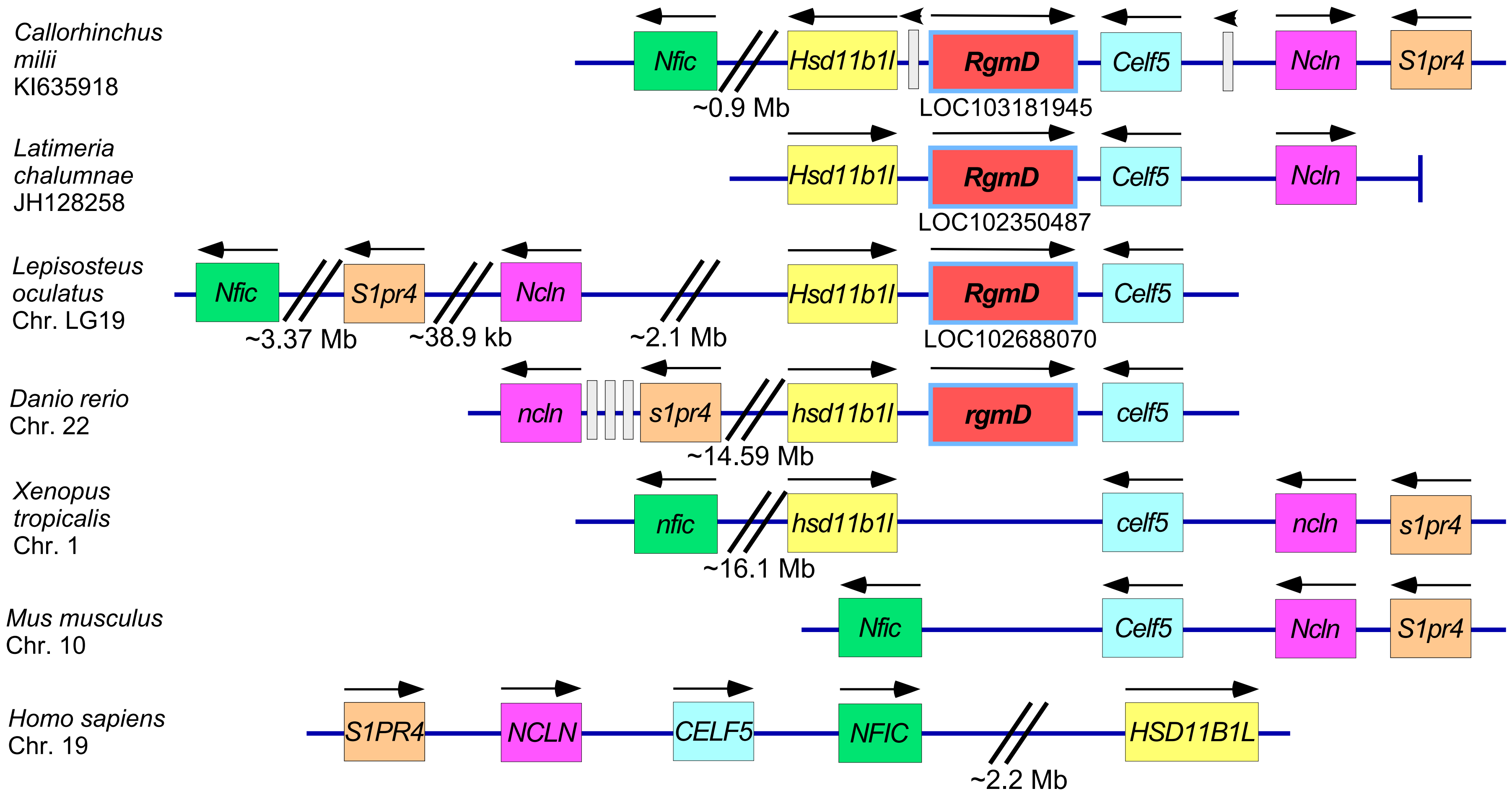
Callorhinchus milii KI635918
Nfic
~0.9 Mb
Hsd11b1l
RgmD
LOC103181945
Celf5
Ncln
S1pr4
Latimeria chalumnae JH128258
Hsd11b1l
RgmD
LOC102350487
Celf5
Ncln
Lepisosteus oculatus Chr. LG19
Nfic
~3.37 Mb
S1pr4
~38.9 kb
Ncln
~2.1 Mb
Hsd11b1l
RgmD
LOC102688070
Celf5
Danio rerio Chr. 22
ncln
s1pr4
~14.59 Mb
hsd11b1l
rgmD
celf5
Xenopus tropicalis Chr. 1
nfic
~16.1 Mb
hsd11b1l
celf5
ncln
s1pr4
Mus musculus Chr. 10
Nfic
Celf5
Ncln
S1pr4
Homo sapiens Chr. 19
S1PR4
NCLN
CELF5
NFIC
~2.2 Mb
HSD11B1L
