# Supplementary material for: Gene expression in notochord and nuclei pulposi: a study of gene families across the chordate phylum
Source: BMC Ecol Evol. 2023 Oct 27;23:63. doi: 10.1186/s12862-023-02167-1 (PMC10605842; doi:10.1186/s12862-023-02167-1)
Supplement: Supplementary file 6 — Additional file 6: Figure S6. Comparative view of the genomic context of hemichordate, tunicate and vertebrate Coro7 genes. [file 12862_2023_2167_MOESM6_ESM.pdf]

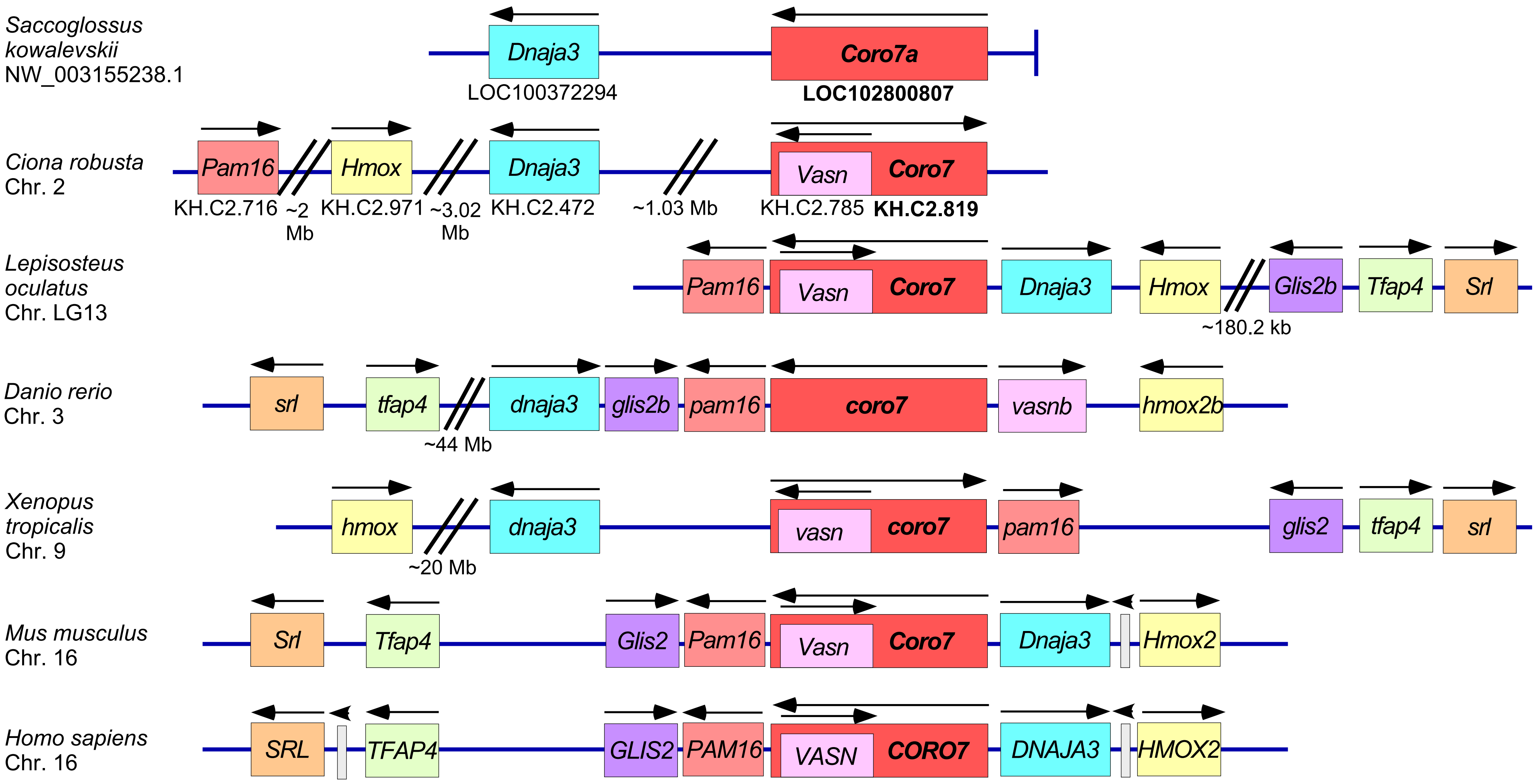

Saccoglossus kowalevskii NW_003155238.1
Dnaja3
Coro7a
LOC100372294
LOC102800807
Ciona robusta Chr. 2
Pam16
Hmox
Dnaja3
Vasn
Coro7
KH.C2.716
~2 Mb
KH.C2.971
~3.02 Mb
KH.C2.472
~1.03 Mb
KH.C2.785
KH.C2.819
Lepisosteus oculatus Chr. LG13
Pam16
Vasn
Coro7
Dnaja3
Hmox
Glis2b
Tfap4
Srl
~180.2 kb
Danio rerio Chr. 3
srl
tfap4
dnaja3
glis2b
pam16
coro7
vasnb
hmox2b
~44 Mb
Xenopus tropicalis Chr. 9
hmox
dnaja3
vasn
coro7
pam16
glis2
tfap4
srl
~20 Mb
Mus musculus Chr. 16
Srl
Tfap4
Glis2
Pam16
Vasn
Coro7
Dnaja3
Hmox2
Homo sapiens Chr. 16
SRL
TFAP4
GLIS2
PAM16
VASN
CORO7
DNAJA3
HMOX2
